# Acupuncture influences multiple diseases by regulating gut microbiota

**DOI:** 10.3389/fcimb.2024.1371543

**Published:** 2024-07-08

**Authors:** Huimin Xu, Yingzhe Luo, Qiaoqi Li, Hong Zhu

**Affiliations:** ^1^ Department of Abdominal Tumor Multimodality Treatment, Cancer Center, West China Hospital, Sichuan University, Chengdu, Sichuan, China; ^2^ Acupuncture and Tuina School, Chengdu University of Traditional Chinese Medicine, Chengdu, Sichuan, China; ^3^ Department of Oncology, Hospital of Chengdu University of Traditional Chinese Medicine, Chengdu, Sichuan, China

**Keywords:** acupuncture, gut microbiota, metabolic disease, gastrointestinal disease, mental disorders, nervous system disease

## Abstract

Acupuncture, an important green and side effect-free therapy in traditional Chinese medicine, is widely use both domestically and internationally. Acupuncture can interact with the gut microbiota and influence various diseases, including metabolic diseases, gastrointestinal diseases, mental disorders, nervous system diseases, and other diseases. This review presents a thorough analysis of these interactions and their impacts and examines the alterations in the gut microbiota and the potential clinical outcomes following acupuncture intervention to establish a basis for the future utilization of acupuncture in clinical treatments.

## 1 Introduction

Gut microbiota, an important microbial ecosystem in the human body, comprises approximately 80% of the total microbial population in the human body (Hou et al., 2022). It consists of six major phyla, including *Firmicutes, Bacteroidetes, Actinobacteria, Proteobacteria, Fusobacteria*, and *Verrucomicrobia* (Hou et al., 2022). Gut microbiota plays a crucial role in maintaining the integrity of the intestinal mucosal barrier and gut-brain axis. On the one hand, the gut microbiota and its primary metabolites directly contribute to protecting the integrity of the intestinal epithelial barrier, enhancing the expression of tight junction proteins, and influencing mucosal permeability by regulating cell growth and differentiation. On the other hand, it can also indirectly ensure the normal functioning of an organism’s life activities through the gut-brain axis or the gut neuroendocrine-immune network (Sharma et al., 2010; Marchesi et al., 2016).

Gut microbiota dysbiosis refers to disruption of the intestinal environment caused by various factors that can lead to the occurrence and development of many diseases (Ruff et al., 2020). In gut microbiota dysbiosis, pathogenic bacteria competitively bind to receptors on the surface of intestinal epithelial cells, producing endotoxins and also damage the biological and mechanical barriers of the intestinal mucosa. This triggers mucosal inflammatory reactions, impairing the secretory function of secretory immunoglobulin A through the inherent layer of the mucosa, thereby compromising the intestinal immune barrier (Zyrek et al., 2007; Jones and Versalovic, 2009). Studies have shown that when the intestinal environment is disrupted, various active substances released by pathogenic bacteria and their antigens can cause gastrointestinal diseases (Han et al., 2023). Gut microbiota dysbiosis can affect the metabolism of substances, such as choline and bile acids, as well as energy absorption, thereby leading to metabolic diseases, such as obesity and diabetes (Janssen and Kersten, 2015). It can also lead to the development of cardiovascular diseases by affecting cholesterol metabolism, oxidative stress response, and inflammatory reactions (Stancu et al., 2014; Kelly et al., 2016). Furthermore, dysbiosis of the gut microbiota and its metabolites can affect the normal physiological functions of neurons and the blood-brain barrier (BBB) in the central nervous system, leading to behavioral and emotional abnormalities and, ultimately, central nervous system diseases (Ma et al., 2019).

Acupuncture is a part of traditional Chinese medicine and is one of the most popular forms of alternative and complementary medicine in the world. Acupuncture works by stimulating specific points on the body’s surface, known as acupoints, to produce therapeutic effects. The main methods of stimulation include manual acupuncture, electroacupuncture, and transcutaneous electrical acupoint stimulation (Wen et al., 2021). In different disease states, acupuncture can effectively regulate gut microbiota (**Tables 1**, **2**). According to existing research, acupuncture can balance the abundance and composition of gut microbiota, thus regulating the quantity and ratio of beneficial bacteria and pathogens in the host body. For example, acupuncture can treat irritable bowel syndrome (IBS) effectively by adjusting the ratio of beneficial to pathogenic bacteria and inhibiting the expression of inflammatory factors (Mengzhu et al., 2022). Acupuncture also improves gut microbiota dysbiosis caused by depression by regulating the secretion of beneficial bacteria and metabolites in the body (Qiu et al., 2023). Simultaneously, acupuncture can improve gut microbiota dysbiosis caused by metabolic disorders by restoring the gut microbiota and its metabolites. However, current research on the mechanisms of acupuncture in regulating the gut microbiota is still limited and faces issues of scope and depth (Ding et al., 2023). This review begins with a study of the gut microbiota and systematically summarizes the animal and clinical evidence of acupuncture treatment for different diseases, providing a robust theoretical foundation for the use of acupuncture in the prevention and treatment of related illnesses.

**Table 1:** Acupuncture regulates gut microbiota in animal models.

Bioassay model	Acupoint	Key finding	References
IBS mice	ST25, ST36	*Bacteroidetes, Lactobacillus, Candidatus saccharimonas, Dubosiella↑, Firmicutes, Ileibacterium, Dorea↓*	(Mengzhu et al., 2022)
CUMS mice	GV20, GV29	*Lactobacillus, Lachnospiraceae_NK4A136_group, Bacillus, Desulfovibrio, Candidatus_Saccharimonas↑, Staphylococcus↓*	(Qiu et al., 2023)
Obese Zucker Diabetic Fatty rats	GB26, RN12, ST36, Fenglong (ST40)	*Bacteroidetes, Monoglobus, Marvinbryantia, Adlercreutzia, UCG-005, Coriobacteriales, Faecalibaculum, Muribaculaceae, Ruminococcus↑, Firmicutes, Peptostreptococcaceae, Bifidobacterium↓*	(Ding et al., 2023)
UC mice	CV4, ST36	*Lactobacillus, Lachnospiraceae*↑, *Odoribacter*, *Allobaculum, Streptoccis*↓	(Wei et al., 2019)
UC mice	ST36	*Bacteroidales, Lactobacillus reuteri↑, Firmicutes, Turicibacterales, Erysipelotrichales, Clostridium ruminantium↓*	(Liu et al., 2020)
Chronic colitis mice	ST36	*Bacteroidetes, norank_Muribaculaceae, Roseburia, Faecalibacterium, Bifidobacterium↑, Firmicutes, Proteobacteria, Escherichia-Shigella, Erysipelatoclostridium↓*	(Wang et al., 2020)
FC mice	ST25, ST37	*Bacteroidetes↑, Firmicutes, Proteobacteria↓*	(Xu et al., 2021)
FC mice	ST25, ST37	*Thaumarchaeota, Gemmatinoadetes, Actinobacteria, Rokubacteria, Acidobacteria, Chloroflexi, Euryarchaeota, Tenericutes, Staphylococcaceae↑, Bactteroidetes, Epsilonbacteraeota, Muribaculaceae, Enterobacteriaceae↓*	(Xu et al., 2023)
CAG rats	ST36	*Oscillospirales, Romboutsia, Christensenellaceae↑, Desulfobacterota, Helicobacter↓*	(Huang et al., 2022)
CUMS rats	GV23, PC7	*Firmicutes*↑, *Bacteroidetes↓*	(Li et al., 2021)
CUMS rats	GV16, GV23	*Clostridia, Bacteroidia↑, Bacilli↓*	(Chen et al., 2023)
PSD rats	DU20, DU24	*Lactobacillaceae, Lachnospiraceae↑, Muribaculaceae, Peptostreptococcaceae, Clostridiaceae↓*	(Cai et al., 2023)
Insomnia mice	DU20, SP6, Shenmen (HT7)	*Lactobacillus*↑, *Clostridium XlVb, Lachnospiracea incertae sedis, Anaerovorax, Oscillibacter, Pseudoflavonifractor, Acetatifactor*↓	(Hong et al., 2020)
Obese mice	ST36, ST25, RN4, RN12	*Ruminiclostridium, Bacteroidia, Lachnospiraceae_FCS020↑, Bacteroides_vulgatus↓*	(Xia et al., 2022)
Obese mice	ST25, CV4, ST36, SP6	*Candidate_dibision WWE3, Acidobacteria, Cyanobacteria, Basidiomycota, Blastocladiomycota, Chlamycota, Glomeromycota↑, Fusobacteria, Firmicutes, Spirochmycetes, Thermotogae, Fibrobacteres, Deferribacteria↓*	(Si et al., 2018)
Obese rats	GB26	*Bacteroidota, Cyanobacteria, Prevotella_9↑, Firmicutes↓*	(Wang et al., 2019)
Obesity mice	CV4, ST25, ST36, SP6	*Proteobacteria, Enterobacter, Stenotrophomonas, Helicobacter↑, Firmicutes, Cyanobacteria, Bacteroidetesin, Lachnospiraceae-NK413↓*	(Dou et al., 2020)
T2DM mice	ST36	*Firmicutes*, Lachnoclostridium, Lachnospiraceae_UCG‐006, Odoribacter, Oscillibacter, Desulfovibrio*↓*	(An et al., 2022)
T2DM mice	RN12, ST36	*Firmicutes↑, Bacteroidetes, Eubacterium↓*	(Wang et al., 2022)
NAFLD rats	GB26	*Bacteroidales_S24–7_group, Prevotellaceae, Bacteroidaceae, Blautia, norank_f_Bacteroidales_S24–7_group, Bacteroides, Prevotella_9↑, Ruminococcaceae_UCG-014↓*	(Wang et al., 2023)
PD rats	GB34, ST36	*Alistipes, Vallitalea*, *Lachnoclostridium*, *Pseudoclostridium, Bacteroides*xylanolyticus*, Clostridium, Vallitalea*, *aerotolerans*, *Pseudoclostridium* thermosuccinogenes*, Roseburia* faecis*↑, Bacteroides, Parabacteroides↓*	(Jang et al., 2020)
PD mice	ST36, GV20	*Lactobacillus, Bacteroides, Ruminococcaceae↑, Erysipelotrichaceae↓*	(Han et al., 2021b)
APP/PS1 mice	GV20, GV29, ST36	*Bacteroidetes↑, Proteobacteria, Escherichia-Shigella↓*	(Hao et al., 2022)
AD mice	GV20, ST36	*Lactobacillus, Bifidobacteria↑, Escherichia coli, Bacteroides fragilis↓*	(He et al., 2021)
SCI rats	ST36	*Campylobacterales*, *Helicobacteraceae*, *Helicobacter* ↑, *Proteobacteria, Gammaproteobacteria*, *Erysipelotrichia, Clostridia, Enterobacteriaceae, Dorea*, *Allobaculum*, *Blautia*↓	(Cheng et al., 2022)
Surgical pain-induced delirium-like rats	DU20, KI1	*Verrucomicrobia, Cyanobacteria, Lactobacillales, Akkermansia*↓	(Yang et al., 2022)
Myocardial ischemia reperfusion injury mice	PC6	*Firmicutes, Clostridia↑, Teneticutes, Molliutes, Desulfovibrio, Allobaculum↓*	(Bai et al., 2021)
Middle-cerebral artery occlusion/reperfusion rats	Quchi (LI11), GB34, Housanli	*Firmicutes*, *Lactobacillus, Bifidobacterium↑, Verrucomicrobiota, Escherichia-Shaigella↓*	(Xian et al., 2022)
Ischemic stroke mice	GV20, ST36	*Firmicutes, Verrucomicrobia, Akkermansia↑, Proteobacteria↓*	(Zhang et al., 2023)
Osteosarcoma tumor-burdened mice	Shenshu (BL23), DU20, ST36	*Bacteroidetes↑, Firmicutes, Candidatus Saccharibacteria*↓	(Xu et al., 2020)
Cancer-related fatigue rats	ST36, SP6, CV4, CV6, GV20	*Firmicutes*, *Bacteroidetes*, *Patescibacteria*, Lactobacillus*, Muribaculaceae_unclassified*, *Candidatus_Arthromitus*, Clostridia*_UCG-014_unclassified*↑, *Proteobacteria, Burkholderia-Caballeronia-Paraburkholderia*, *Escherichia-Shigella*, *Streptococcus*↓	(Lv et al., 2022)
Colitis mice	ST36	*Firmicutes, Corynebacteriales, Betaproteobacteriales, Nocardiaceae, Burkholderiaceae, Lachnospiraceae↑, Proteobacteria, Gammaproteobacteria↓*	(Liu et al., 2022)
Obese KOA rats	ST36, GB34, Liangqiu (ST34), Dubi (ST35), Xuehai (SP10)	*Firmicutes, Clostridium, Akkermansia, Butyricimonas, Lactococcus↑, Proteobacteria, Verrucomicrobia↓*	(Xie et al., 2020)
Stress urinary incontinence rats	BL23, Huiyang (BL35)	*Blautia, Prevotellaceae_NK3B31_group, Lachnoclostrium↑, Akkermansia, Lachnospiraceae_NK4A136_group, Parabacteroides↓*	(Li et al., 2022a)
Premature Ovarian failure mice	CV4, ST36, SP6	*Actinobacteria↑, Tenericutes, Alistipes, Pantoea, Rikenellaceae_RC9_gut_group, Ruminococcaceae_UCG-009↓*	(Geng et al., 2022)
APP/PS1 mice	DU20, Hegu (LI4), Feishu (BL13), Pishu (BL20), BL23, ST36	*Christensenellaceae, Clostridiaceae, Prevotellaceae, Rikenellaceae, Bacteroidaceae↑, Porphyromonadaceae, Coliobacteriaceae, Helicobacteraceae, Burkholderiaceae↓*	(Yang et al., 2022b)
APP/PS1 mice	GV20, Yintang (GV29), ST36	*Bacteroidota↑, Proteobacteria, Escherichia-Shigella↓*	(Zhang et al., 2022b)
AD mice	GV20, GV29	*Deltaproteobacteria, Epsilonproteobacteria, Deferribacteres↑, Clostridia↓*	(Jiang et al., 2021)
Peptic ulcer disease mice	Shousanli (LI10), ST36	*Bacteroidetes, Enterorhabdus, norank_f_Muribaculaceae, Lachnospiraceae_NK4A136_group, norank_f_Lachnospiraceae, Lachnoclostridium, Roseburia↑, Firmicutes↓*	(Li et al., 2022b; Li et al., 2022c)
Middle cerebral artery occlusion rats	LI11, ST36	SCFAs-producing bacteria↑	(Ke et al., 2022)
T2DM mice	BL13, BL20, BL23, LI4, Taichong (LR3), ST36, SP6	*Lactobacillus, Blautia↑, Alistipes, Helicobacter, Prevotella↓*	(Zhang et al., 2021)
Irritable bowel syndrome rat	ST25, ST37	*Flavobacteriaceae, Acinetobacter, Arthrobacter, Empedo-bacter, Facklamia Jeotgalicoccus, Sporobacter↑, Bdellovibrionales, Fusobacteriales, Chitinophagaceae, Bilophila, Enterococcus, Fusobacterium, Vam- pirovibrio↓*	(Song et al., 2020)

"↑" means the relative abundance increasing; "↓" means the relative abundance decreasing.

**Table 2:** Changes of gut microbiota in clinical subjects regulated by acupuncture.

Bioassay model	Acupoint	Key finding	References
CD patients	CV12, ST37, SP6, Gongsun (SP4), LR3, Taixi (KI3), LI4, LI11, ST36, ST25	*Bacilli, Lactobacillales Enterococcaceae, Mollutes, RF39*, Bγ*-Proteobacteria, Pastheurellaceae, Pasteurellales*↑	(Bao et al., 2022)
Antipsychotic-related constipation patients	ST25, SP14, ST37	*Gammaproteobacteria, Enterobacteriales, Enterobacteriaceae, Klebsiella↑, Megasphaera, Burkholderiaceae, Succinivibrio↓*	(Zheng et al., 2021)
Hypertensive patients	LR3, KI3, Renying (ST9), PC6	*Bacteroidetes, Bifidobacteriaceae, Blautia↑, Firmicutes, Escherichia-Shigella↓*	(Wang et al., 2021)
KOA patients	ST35, Neixiyan (EX-LE5), Ququan (LR8), Xiyangguan (GB33), Ashi point	*Blautia, Streptococcus, Eubacteriumj_hallii_group↑, Bacteroide, Agathobacter, Aeromonadales↓*	(Wang et al., 2021)

"↑" means the relative abundance increasing; "↓" means the relative abundance decreasing.

## 2 Effect of acupuncture on gut microbiota in different diseases

### 2.1 Gastrointestinal disease

#### 2.1.1 Ulcerative colitis (UC)

UC, classified as an inflammatory bowel disease (IBD), displays symptoms, including abdominal discomfort, mucous stools, and bloody diarrhea (Bressler et al., 2015). Recent research has indicated that an imbalance in the gut microbiota, including changes in its composition and abundance, is closely associated with the occurrence, progression, and outcomes of UC (Cani and Knauf, 2016; Lin and Zhang, 2017). Electroacupuncture promotes the recovery of colonic mucosa, causing changes in the gut microbiota in the body, and significantly improves the severity of UC (Wei et al., 2019; Liu et al., 2020; Wang et al., 2020a). Wei et al. found that *Bacteroides*, *Odoribacter*, *Allobaculum*, and *Streptococcus* in UC mice significantly increased, while *norank_f_Bacteroidales_S24–7_group*, *Lactobacillus*, and *Lachnospiraceae_UCG-001* significantly decreased. After continuous electroacupuncture stimulation of the Guanyuan (CV4) point and Zusanli (ST36) point for 15 minutes each time for 5 days (rarefaction wave, frequency 2/15 Hz, and current strength 0.4–0.6mA), electroacupuncture inhibited *Odoribacter*, *Allobaculum*, and *Streptococcus*, promoted *Lactobacillus* and *Lachnospiraceae* (including *Lachnospiraceae_NK4A136_group* and *unclassified_f_Lachnospiraceae*) (Wei et al., 2019). These bacteria played an important role in restoring the gut microbiota stability of UC mice through electroacupuncture therapy. It has been reported that *Lactobacillus*, *Lachnospiraceae*, and *Streptococcus* have been proven to alleviate or promote UC (Liu et al., 2011; Kojima et al., 2012; Machiels et al., 2017). It has been well known that the gut microbiota can affect T cell function and modulate the Regulatory T cells (Treg)/T helper 17 cells (Th17) axis (Ivanov et al., 2008; Atarashi et al., 2011; Omenetti and Pizarro, 2015). Tregs and Th17 represent distinct subsets of CD4^+^ T cells crucially involved in the pathogenesis and progression of UC. Th17, as a subset of T-helper cells, is known for promoting tissue inflammation, while Treg is identified as inhibiting various inflammation and immune responses (Omenetti and Pizarro, 2015). In UC mice, the Treg subtype decreases while the Th17 subtype increases (Sun et al., 2017). In the findings of Wei et al., microbial diversity decreased with the increase of Treg cells and decreased with the decrease of Th17 cells (Wei et al., 2019). Electroacupuncture treatment promoted an increase in the diversity and abundance of gut microbiota in UC mice, which was positively correlated with the improvement of Treg cells (Wei et al., 2019). After continuous 8 days of 15-minute electroacupuncture stimulation (2 Hz, 1mA) on ST36, the levels of pro-inflammatory mediators, including C-reactive protein (CRP), interferon-gamma (IFN-γ), tumor necrosis factor α (TNF-α), and interleukin (IL)-6 induced by dextran sulfate sodium (DSS) were significantly inhibited in the UC model through the myeloid differentiation primary response gene 88 (MyD88)-dependent pathway of toll-like receptor 4 (TLR4) signaling, improving intestinal mucosal barrier function, promoting the relative abundance of *Lactobacillus reuteri* and *Lactobacillus vaginalis*, and inhibiting the relative abundance of *Clostridium ruminantium*, thereby improving DSS-induced colitis (Liu et al., 2020a). Wang et al. then performed low-frequency (10Hz, 1mA) and high-frequency (100Hz, 1mA) electroacupuncture treatment on UC mice for 30 minutes each day for up to 56 days. Compared to normal mice, the relative abundances of *no-rank Muribaculaceae*, *Roseburia*, *Faecalibacterium*, and *Bifidobacterium* were significantly reduced in DSS mice. The abundance of *Muribaculaceae* and *Roseburia* in the low-frequency electroacupuncture and high-frequency electroacupuncture groups increased, while the abundance of *Faecalibacterium* and *Bifidobacterium* improved only in the high-frequency electroacupuncture group (Wang et al., 2020a). It has been reported that reducing genera that produce butyrate, such as *Faecalibacterium* and *Roseburia*, and increasing pathogenic *Escherichia coli* strains in colitis can lead to intestinal barrier defects, while supplementation with specific *Bifidobacterium* and *Lactobacillus* strains can upregulate TJs and E-Cadherin and reduce intestinal permeability (Dai et al., 2012; Ren et al., 2017; Chen et al., 2020). In addition, compared to the control group, the DSS group showed increased abundance of *Escherichia-Shigella* and *Erysipelatoclostridium*, while these values decreased in both low-frequency electroacupuncture and high-frequency electroacupuncture groups compared to the DSS group. The high-frequency electroacupuncture treatment and the microbiota from both low-frequency electroacupuncture treatment and high-frequency electroacupuncture treatment can activated the extracellular signal-regulated kinase (ERK) 1,2/c-Jun amino-terminal kinases (JNK)/p38 mitogen-activated protein kinase (MAPK) signaling pathway (Wang et al., 2020a). Previous studies have shown that the MAPK signaling pathway can mediate the barrier-protective effects of probiotics (Dai et al., 2012). Therefore, electroacupuncture treatment can promote the MAPK signaling pathway by modulating the gut microbiota, thereby maintaining the integrity of the intestinal barrier.

#### 2.1.2 Crohn’s disease (CD)

**Figure 1:**
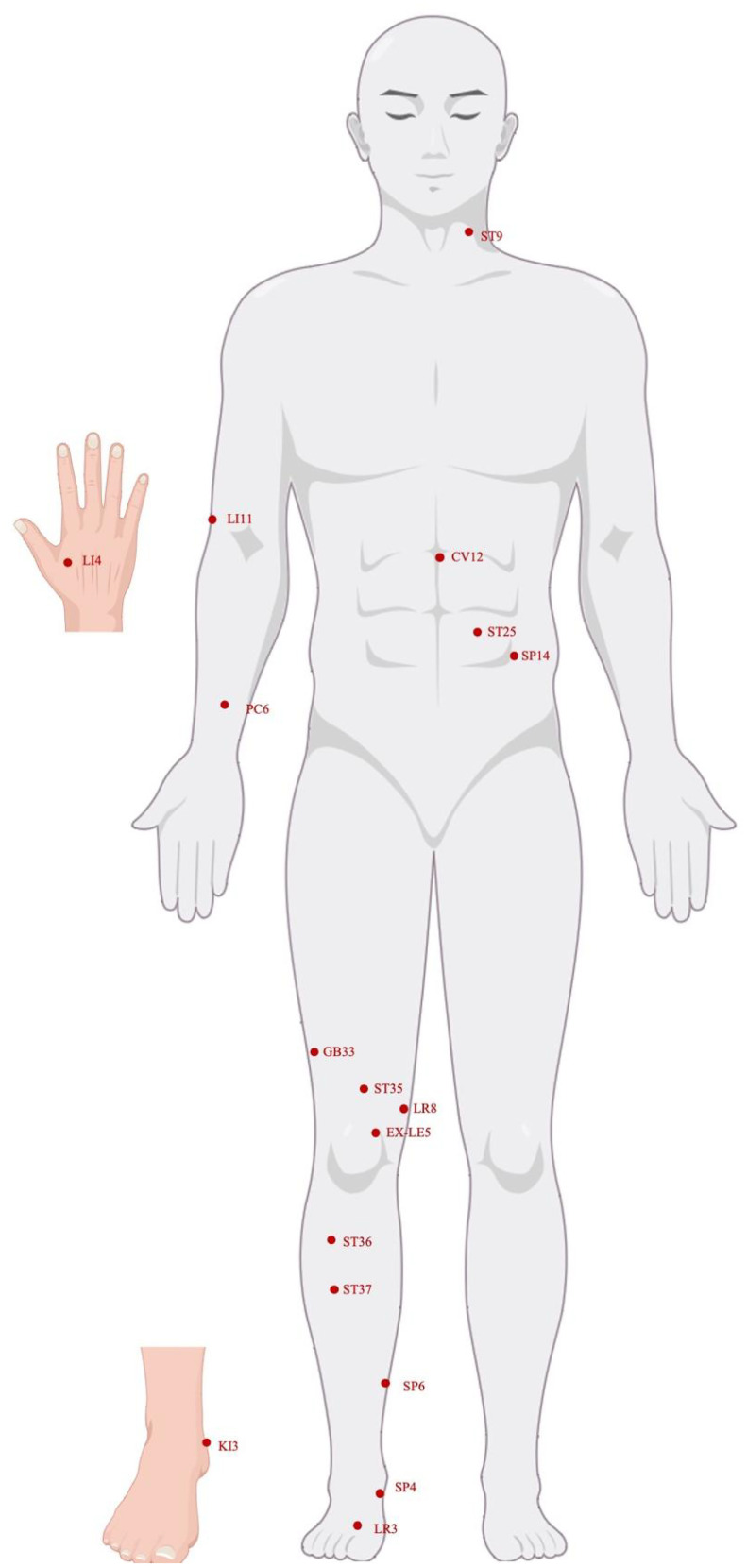
Human acupuncture model photo. By Figdraw.

CD is a chronic, recurrent inflammatory disorder that manifests as abdominal pain, diarrhea, and weight loss (Torres et al., 2017). Recent investigations have revealed considerably reduced gut microbiota and immune responses in individuals with active CD compared with their healthy counterparts. The active phase of the disease is marked by alterations in the diversity of the gut microbiota, including a reduction in obligate anaerobes and an increase in facultative anaerobes, such as *Escherichia coli* (Lloyd-Price et al., 2019). Moreover, evidence indicates that damage to the integrity of the intestinal epithelial barrier and the resulting increase in intestinal permeability leads to the infiltration of gut microorganisms and antigens, provoking abnormal immune responses in individuals with CD (Geremia et al., 2014). Thus, the gut microbiota and inflammation are considered key factors in the development of CD (Zhang and Li, 2014; Kaplan and Ng, 2017), and acupuncture has been shown to modulate them (Qi et al., 2018; Wei et al., 2019). Findings from a clinical trial have shown that acupuncture treatment 3 times a week for 12 weeks boosted the number of operational taxonomic units, ACE index, and the proportion of bacteria (*Roseburia faecis*, *Roseburia*, *Coprococcus*, and *Lachnospira*) that produce short-chain fatty acids (SCFAs) and anti-inflammatory bacteria (**Figure 1**) (Bao et al., 2022). The composition of the participants’ microbiota also tended to resemble that of healthy individuals (Bao et al., 2022). These findings suggest that acupuncture may contribute to the restoration of a balanced gut microbiota. Acupuncture can effectively lower the levels of diamine oxidase, lipopolysaccharide (LPS), and Th1/Th17-related cytokines in the blood, leading to enhanced intestinal barrier function and reduced secretion and migration of pro-inflammatory cytokines in the intestine, thereby improving disease activity in patients with mild to moderately active CD (Bao et al., 2022). Substantial evidence suggests that gut inflammation can induce alterations in microbiota composition, as observed in animal models of colitis and infectious gastroenteritis (Ni et al., 2017). Therefore, the amelioration of CD symptoms by acupuncture may be attributed to an increased abundance of anti-inflammatory and SCFAs-producing bacteria, restoration of the intestinal epithelial barrier, and suppression of the production and release of pro-inflammatory cytokines related to Th1/Th17 signaling in the intestines.

#### 2.1.3 IBS

IBS is a gastrointestinal disorder characterized by abdominal pain and changes in bowel habits, with an estimated prevalence of 10–20% (Sultan and Malhotra, 2017). A proven connection between the gut microbiota and IBS has revealed that the gut microbiota can activate the immune system, break down substances, and generate essential nutrients to maintain the physiological functions of the host ecosystem. However, differences in gut microbiota diversity, richness, and composition have been identified between patients with IBS and healthy controls (Maharshak et al., 2018). Gut microbiota dysbiosis is associated with the symptoms of IBS. Studies have found that transferring the gut microbiota from animals or patients with IBS to healthy individuals induces the development of IBS symptoms (Crouzet et al., 2013; Sultan and Malhotra, 2017). Electroacupuncture stimulation at Tianshu (ST25) and ST36 for 15 minutes (frequency 2/15 Hz, 0.5mA) a day for seven consecutive days alleviates visceral hypersensitivity induced by IBS (Mengzhu et al., 2022). Sun et al. found that electroacupuncture increased the richness and diversity of the gut microbiota compared with the IBS model group in a 16S rRNA gene sequencing analysis. Moreover, the *Firmicutes/Bacteroidetes* ratio decreased after electroacupuncture treatment (Mengzhu et al., 2022). The *Firmicutes/Bacteroidetes* ratio indicates alterations in the gut bacteria population and has been reported to be elevated in patients with IBS (Tap et al., 2017). Electroacupuncture therapy also notably increased the relative abundance of *Lactobacillus* in the IBS model group; *Lactobacillus* has been widely used to treat IBS and has been shown to effectively alleviate visceral hypersensitivity (Ait-Belgnaoui et al., 2018; Preston et al., 2018). This suggests that electroacupuncture alleviates visceral hypersensitivity in patients with IBS by modulating lactobacilli. Recently, the connection between the gut, brain, and microorganisms in the body has gained significant attention. The link between gut health, brain function, and microbiome is essential for maintaining homeostasis and for controlling gastrointestinal motility, sensation, autonomy, and secretory functions (Burokas et al., 2015). One study found that compared with a normal group, an IBS animal model group exhibited significantly higher expression of corticotropin-releasing factor (CRF) in colonic tissue, which was reduced after electroacupuncture treatment (Mengzhu et al., 2022). CRF is considered a major mediator of the brain-gut axis and is a pivotal factor in the progression of IBS (Tache et al., 2018). These findings suggest that electroacupuncture may alleviate IBS by regulating CRF expression. Electroacupuncture alleviates IBS and modulates gut microbiota dysbiosis and CRF expression (Mengzhu et al., 2022). Hence, the factors that lead to the effect of electroacupuncture on IBS may be related to the interaction between the microbiota, gut, and brain. Nevertheless, further investigations are necessary to delve deeper into the association between the microbiota-gut-brain axis and the influence of electroacupuncture on IBS.

#### 2.1.4 Functional constipation (FC)

Individuals with FC often experience feelings of tension and incomplete evacuation; however, the exact pathogenesis remains unclear (Aziz et al., 2020; Bharucha and Lacy, 2020). Recently, differences in the composition of the gut microbiome have been observed between individuals with FC and healthy individuals (Parthasarathy et al., 2016). Mice that received fecal microbiota transplantation from patients with constipation displayed reduced gastrointestinal motility and exhibited typical symptoms of constipation, indicating that abnormal gut microbiota in patients with constipation may reduce intestinal motility (Ge et al., 2017). Consequently, it may have additional negative effects on intestinal bacteria, setting off a harmful loop of imbalance in the gut and intestinal movement problems, ultimately leading to chronic or worsening diseases. Current research indicates that electroacupuncture therapy can effectively restore the balance of intestinal microbiota in constipated mice, promote intestinal transit rate, increase fecal pellets, and therefore improve constipation symptoms. Jin et al. selected Ci Liao (BL32), Zhong Liao (BL33), and Xia Liao (BL34) for 14 days of 30-minute (frequency 2–15Hz, 1–1.5mA) electroacupuncture treatment on slow transit constipation (STC) rats (Jin et al., 2020). Compared to the model group, the number of fecal pellets increased in the electroacupuncture group within 24 hours. Although the fecal pellet volume was lower than the mosapride group, it was not significantly different from the *Bacillus licheniformis* group. Electroacupuncture therapy increased the intestinal transit rate in STC rats. At the same time, electroacupuncture significantly increased the abundance of *Bacteroides*, *Parabacteroides*, *Prevotella*, and *Paraprevotella*. Compared to normal rats and STC rats untreated with drugs (including *Bacillus licheniformis* and mosapride), the abundance of potential pathogens such as *Clostridium* XI and *Ruminococcus* in STC rats decreased (Jin et al., 2020). Xu et al. selected ST25 and Shangjuxu (ST37) for 10 sessions of 30-minute (frequency of 3/15Hz, 1mA) electroacupuncture stimulation on STC mice (Xu et al., 2021b). In the STC group, the abundance of *Firmicutes* and *Proteobacteria* increased significantly, while *Bacteroidetes* decreased significantly. Electroacupuncture therapy reduced the abundance of *Firmicutes* and *Proteobacteria*, and increased *Bacteroidetes* (Xu et al., 2021b). The ratio of *Firmicutes* to *Bacteroidetes* (F/B ratio) is commonly used to assess intestinal health (Mancabelli et al., 2017). The F (46.6%)/B (47.4%) ratio in the STC group was close to 1/1, while the F (44.68%)/B (51.75%) ratio in the electroacupuncture group was similar to the NC group (Xu et al., 2021b). Dominant bacteria such as *Roseburia*, *Turicibacter*, and *Lachnoclostridium* in the STC group decreased significantly in the electroacupuncture group. Electroacupuncture treatment restored the composition of gut microbiota in constipated mice to a level similar to that of healthy mice. In another experiment involving antibiotic-induced disruption of gut microbiota, further evidence confirmed the improvement of constipation symptoms through electroacupuncture therapy. Mice in the antibiotic group and the control group were both subjected to 10 sessions of 30-minute (frequency of 3/15Hz and intensity of 1mA) electroacupuncture stimulation on ST25 and ST37. The results showed that all constipation symptoms significantly improved after electroacupuncture treatment, but the effect of electroacupuncture was eliminated after antibiotic feeding (Xu et al., 2023). Therefore, it is speculated that the positive effect of electroacupuncture on intestinal motility would be reduced in the absence of gut microbiota. Accumulating evidence suggests that the gut microbiota directly regulate intestinal motility through the enteric nervous system (ENS) or indirectly through the intestinal immune system (Kashyap et al., 2013; Grasa et al., 2015; Cao et al., 2017). To more accurately clarify the potential mechanisms by which intestinal microbiota mediated by electroacupuncture resolve constipation, further exploration is needed on how changes in the gut microbiota and its metabolites induced by electroacupuncture regulate intestinal motility through the ENS and the intestinal immune system.

#### 2.1.5 Antipsychotic-related constipation

Constipation caused by antipsychotic medication is a common issue that many people face (Rege and Lafferty, 2008). Constipated patients received 4 weeks of electroacupuncture treatment, with 12 sessions of 30-minute (frequency of 2/15Hz, 0.1–1mA) electroacupuncture stimulation at acupoints ST25, Fujie (SP14), and ST37. Electroacupuncture treatment for antipsychotic-related constipation was more effective than the use of sham electroacupuncture, and the therapeutic effect of electroacupuncture was sustained. The sustained effects of electroacupuncture treatment and shallow needling were superior to the therapeutic effect of lactulose (Wu et al., 2014). In the study by Zheng et al., a correlation was found between antipsychotic-related constipation in patients aged 24–55 and the abundance of *Parabacteroides* (**Figure 1**) (Zheng et al., 2021). *Parabacteroides* are potential probiotics, and differences in these levels were observed in children with chronic functional constipation (de Meij et al., 2016; Chang et al., 2019). Electroacupuncture treatment affected *Parabacteroides*, which may be the reason for the anti-constipation effect in patients with antipsychotic-related constipation aged 24–55. Furthermore, Spearman rank correlation analysis reported that six bacterial taxa including *Coprobacter*, *Corynebacterium*, *Cutibacterium*, *Cupriavidus*, *Enhydrobacter*, and *Parabacteroides* were associated with the severity of constipation, and their abundance after electroacupuncture treatment was opposite to that before treatment (Zheng et al., 2021). However, it is currently unclear which specific bacteria species lead to constipation and further research is needed to evaluate whether specific bacterial species in constipated patients are affected by electroacupuncture treatment.

#### 2.1.6 Chronic atrophic gastritis (CAG)

CAG is a precancerous condition of the stomach (Bockerstett et al., 2020). Disturbances in the gut microbial community have been identified as a significant disease-related factors in CAG development. Yu et al. examined CAG rats and found that the gut microbiota composition changed significantly as gastric mucosal disease progressed from normal tissue injury to gastric cancer. As the level of the microbiota increased, its diversity decreased, the proportion of butyrate-producing bacteria decreased, harmful bacteria, such as *Shigella*, dominated the gut, and different characteristics of the gut microbiota were observed at each stage of the disease (Yu et al., 2020). After four weeks of 30-minute (output parameters were sparse and dense waves, which sparse wave 4Hz and dense wave 50Hz) electroacupuncture stimulation at ST36 in CAG rats, it was found that electroacupuncture regulated the expression levels of p53, c-myc, and Bcl-2 in the gastric mucosa, promoting the repair of gastric mucosal tissue in CAG rats (Huang et al., 2022). Comparisons between CAG and electroacupuncture groups using LEFse analysis showed a reduction in the relative abundance of harmful bacteria, including *Desulfovibrionaceae*, *o_Campylobacterales*, *Desulfobacterota*, *Helicobacteraceae*, *Campylobacter*, *Desulfovibrionia*, *Desulfovibrionales*, and *Helicobacter pylori* in the electroacupuncture group, compared to the CAG group (Huang et al., 2022). This suggests that electroacupuncture treatment effectively suppresses the proliferation of these harmful bacteria. AS we all known that *Helicobacter pylori* is a destructive factor in diseases such as peptic ulcers, acute and chronic gastritis, GC, and other gastric mucosal injuries (Smet and Menard, 2020). Enterohepatic *Helicobacter* species not only has pro-inflammatory activity but also causes DNA damage, stimulating the expression of cytokines such as IL-22, IL-17, IFN-γ, IL-6, and inducible nitric oxide synthase (iNOS) (Mengping et al., 2018). Electroacupuncture therapy effectively inhibits the relative abundance of *Helicobacter pylori* suppresses inflammatory reactions, and promotes the repair of gastric mucosal tissues. In the electroacupuncture group, beneficial bacteria, such as *Christensenellaceae*, *Romboutsia*, and *Oscillosporia*, were enriched in the gut microbiota (Huang et al., 2022). The *Christensenellaceae* family, which plays an important role in human health, is found in human feces, colonic mucosa, ileum, and appendix and represents a new branch of the *Firmicutes* phylum (Waters and Ley, 2019). Electroacupuncture may promote the repair of gastric mucosal tissue by regulating gut microbiota, thereby modulating the apoptosis of gastric mucosal epithelial cells.

### 2.2 Mental disorder

#### 2.2.1 Depression

**Figure 2:**
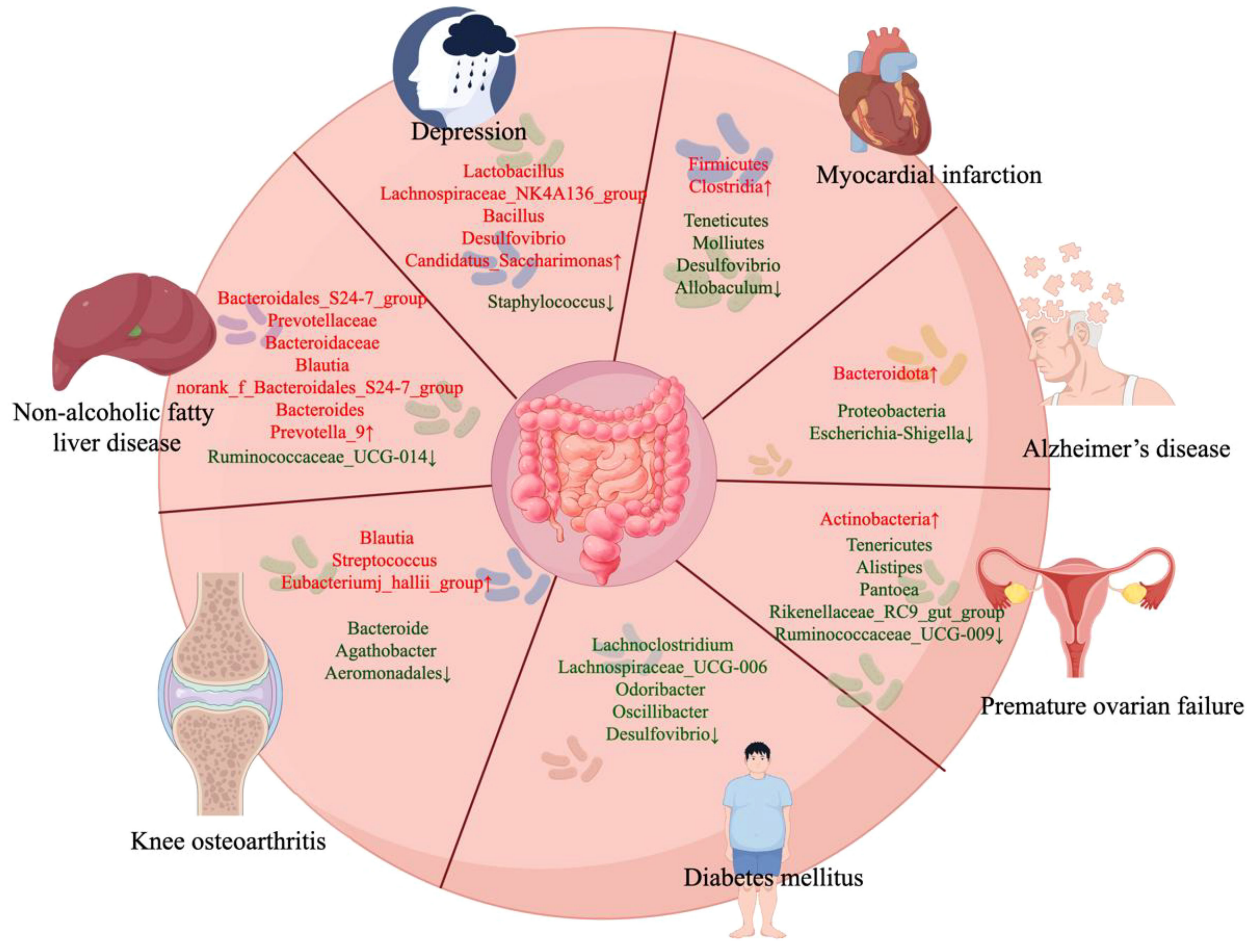
Acupuncture affects the changes of gut microbiota in different diseases. By Figdraw.

Depression is a common mental disorder characterized by heterogeneous symptoms, including persistent low mood, decreased self-esteem and energy, loss of interest, changes in weight, insomnia or hypersomnia, and impaired cognitive functions, such as attention and memory (Organization, 2017). Persistent depression significantly affects daily life, and severe depression may lead to suicide, threatening social stability and development (Fava and Kendler, 2000). Gut microbiota disorders are pivotal factors associated with depression (Rogers et al., 2016). Clinical trials have demonstrated that significant variations in gut microbiota composition between patients with depression and healthy control groups, whereas the transfer of the “depressive microbiota” from patients with major depressive disorder into germ-free (GF) mice has produced similar depressive-like effects compared with “healthy microbiota” form healthy control group (Zheng et al., 2016b). Animal experiments have also shown that with chronic administration, prebiotics, such as fructo-oligosaccharides and galactooligosaccharides, exhibits both antidepressant and anxiolytic effects (Burokas et al., 2017). Acupuncture leads to changes in the gut microbiota, affecting the *Gammaproteobacteria* class, *Ruminococcaceae* genus, and *Lachnoclastridium* genus, which are closely associated with anxiety and depression (Knuesel and Mohajeri, 2021; Simpson et al., 2021). Qiu et al. also found that continuous 14 days electroacupuncture (current 1mA, frequency of 2Hz) exerts antidepressant effects by adjusting the abundance of *Lactobacillus* and *Staphylococcus* (Qiu et al., 2023). After chronic unpredictable mild stress (CUMS) stimulation, the abundance of *Lactobacillus* in mice decreased, whereas that of *Staphylococcus* increased. After electroacupuncture therapy, the abundance of *Lactobacillus* increased to near-normal levels, whereas that of *Staphylococcus* decreased (Qiu et al., 2023) (**Figure 2**). Recent studies have shown that *Lactobacillus* alleviates depression. *Lactobacillus* can improve the protein expression of the norepinephrine, monoamines dopamine, serotonin (5-HT) (which has a negative relationship with depression), and brain-derived neurotrophic factor (BDNF) in mice, thereby improving depressive behavior (Bravo et al., 2011). BDNF is an important neurotrophic factor that is required for the survival and normal function of neurons in the brain (McAllister et al., 1995; Wainwright and Galea, 2013). A previous study showed that disorders of intestinal microorganisms decrease hippocampal neurotransmitter levels, including 5-HT and BDNF (Caviedes et al., 2017; Liu et al., 2020b). Additionally, acupuncture at the Lianquan (CV23) and Daling (PC7) acupoints upregulated BDNF levels in the hippocampal tissue of CUMS mice (Li et al., 2021a). Long-term administration of *Lactobacillus rhamnosus* (JB-1) induces region-dependent changes in GAB1b mRNA in the brain and reduces the expression of Gamma aminobutyric acid (GABA) receptors in the amygdala, hippocampus, and blue spot, thereby exerting antidepressant effects, reducing corticosterone levels, and improving corticosterone-induced anxiety-depressive behavior (Bravo et al., 2011). *Staphylococcus* has been shown to impair the nervous system, with *S. aureus* producing staphylococcal enterotoxins and glutamate, stimulating the vagus nerve (VN) and signaling to the brain, leading to vomiting and nausea (Hu et al., 2007). Another study confirmed that acupuncture could reduce depression-like behaviors in CUMS rats by regulating the gut-liver-brain axis (Chen et al., 2023). CUMS rats treated with acupuncture (20 min) at the Fengfu (GV16) and Shangxing (GV23) acupoints for 4 weeks showed notable improvements in depression-like behavior and elevated the expression of 5-HT (Chen et al., 2023). Therefore, acupuncture may exert antidepressant effects by stimulating the VN and thereby improving the gut microbiota disorders caused by CUMS.

#### 2.2.2 Poststroke depression (PSD)

PSD is the most common neurological and psychiatric consequence of a stroke. PSD is characterized by cognitive impairment, poor rehabilitation outcomes, and increased mortality (Hadidi et al., 2009; Bartoli et al., 2013). Previous studies have shown that the species richness index is decreased in PSD rats (Jiang et al., 2021). A study showed that continuous 20-minute (frequency of 2Hz) electroacupuncture stimulation of Baihui (DU20) and Shenting (DU24) for 14 days not only significantly alleviated depressive behavior in PSD rats, but also significantly altered the gut microbiota phenotype of these rats (Cai et al., 2023). The relative abundance of *Muribaculaceae*, *Peptostreptocccaceae*, *Oscillospiraceae*, *Ruminococcaceae*, and *Clostridiaceae* increased in PSD rats, while *Lactobacilaceae*, *Lachnospiraceae*, and *Bacteroidaceae* decreased. Electroacupuncture treatment reversed the abundance of *Muribaculaceae*, *Lactobacillaceae*, *Lachnospiraceae*, *Peptostreptocccaceae*, and *Clostridiaceae* (Cai et al., 2023). Metabolomic analysis of PSD revealed a close association between its pathogenesis and abnormal lipid metabolism. Cai et al. found that fecal metabolites in PSD rats were involved in pathways related to ovarian steroidogenesis, steroid hormone biosynthesis, chemical carcinogenesis, and plant hormone biosynthesis (Cai et al., 2023). Three out of the four enriched pathways were related to lipid metabolism. Virtanen et al. discovered that low-density lipoprotein cholesterol predicted a reduced risk of long-term depressive symptoms (Virtanen et al., 2017). Another study revealed an association between corticosteroids and increased risk of depression (Weina et al., 2018). Acupuncture treatment effectively alleviated depression by altering hepatic lipid metabolism and reducing leptin insensitivity (Jung et al., 2021). Cai et al. also found that electroacupuncture treatment could regulate steroid-hormone biosynthesis (Cai et al., 2023). It has been speculated that the one potential mechanism by which electroacupuncture treatment alleviates depressive behavior in PSD is through the regulation of gut microbiota and lipid metabolism. However, further mechanistic studies are required to confirm this hypothesis.

#### 2.2.3 Insomnia

Insomnia is the second most common mental disorder (Wittchen et al., 2011). A study reported significantly elevated melatonin levels in the pineal bodies of mice that underwent acupuncture, thereby improve the effect of sleep (Hong et al., 2020).There was higher abundance of *Lactobacillus* and lower abundance of *Lachnospiracea incertae sedis* in the mice after acupuncture treatment compared with the mice with insomnia (Hong et al., 2020). *Lactobacillus* has been shown to decrease the levels of inflammatory cytokines and conflict with TLR-related inflammation in chronic sleep fragmentation mice (Vanuytsel et al., 2014). *Lachnospiracea incertae sedis* can exert anti-inflammatory effects by triggering Tregs, thereby further modulating the immune system (Fang et al., 2017). Furthermore, acupuncture also has been proven to have immunomodulatory effects (Watanabe et al., 2015; Huang et al., 2019). Therefore, acupuncture may exert therapeutic effects on insomnia by regulating the gut microbiota to modulate the host immune response.

### 2.3 Metabolic diseases

Metabolic diseases are a series of interconnected metabolic disorders, including insulin resistance, obesity, non-alcoholic fatty liver disease (NAFLD), and dyslipidemia, which greatly increase the incidence and mortality of cardiovascular diseases, while reducing life expectancy (Dabke et al., 2019; Agus et al., 2021). Abnormal energy metabolism is characterized by changes in metabolic flux and fat deposition at abnormal locations (SantaCruz-Calvo et al., 2022). Disrupted of the microbiota can contribute to various metabolic disorders (Fan and Pedersen, 2021). Accumulating evidence suggests that bioactive metabolites produced by microbial dysbiosis directly regulate the levels of important liver metabolites through the production of bioactive compounds, affecting glucose homeostasis or indirectly disturbing metabolism by being absorbed through the enterohepatic circulation, leading to the accumulation of toxic compounds in the liver (Canfora et al., 2019; Jastroch et al., 2020; Krisko et al., 2020). By modulating oxidative stress, fat deposition in abnormal locations, and metabolic flux, electroacupuncture has the potential to ameliorate abnormal energy metabolism (Ding et al., 2023). Furthermore, electroacupuncture can also cause changes in the gut microbiota of metabolic diseases. Next, we will discuss the changes and effects of electroacupuncture on gut microbiota in metabolic diseases (Vojinovic et al., 2019; Rodriguez et al., 2020; Han et al., 2022; Miao et al., 2022).

#### 2.3.1 Obesity

Global public health is facing a major challenge due to the prevalence of obesity (Caballero, 2019a). Obesity-induced gastrointestinal immune damage disrupts the balance of gut microbiota in the host. Acupuncture at Daimai (GB26) three times a week for 20 minutes per session over 8 consecutive weeks reduced body weight and suppressed body fat accumulation in high-fat diet (HFD) rats (Wang et al., 2019a). Acupuncture also suppressed the elevation of blood lipid levels, including alanine transaminase (ALT), aspartate transaminase (AST), triglyceride (TG) and total cholesterol (TC), induced by HFD in obese rats and improved insulin resistance (Wang et al., 2019a). Furthermore, acupuncture significantly decreased the abundance of *Firmicutes*, increased the abundance of *Bacteroidetes*, and reduced the F/B ratio. Through genus-level acupuncture treatment, there was a significant increase in the relative abundance of *Prevotella_9* (Wang et al., 2019a). In another study, electroacupuncture at ST36, ST25, Guanyuan (RN4), and Zhongwan (RN12) on HFD mice promoted the expression of Defa20, Defa41, and Defa5 in obese mice, inhibited lipid absorption, and improved glucose tolerance, with parameter settings of 2mA 15 Hz (Xia et al., 2022). Human defensin 5 (encoded by Defa5), an antimicrobial peptide abundantly present in the small intestine, plays a crucial role in the defense against enteric pathogens (Salzman et al., 2003). Previous studies have shown that individuals with severe obesity display reduced defensin alpha 5 (Defa5) levels and that there is a negative correlation between serum lysozyme levels and body mass index (Hodin et al., 2011). Recent research found that levels of intestinal defensins (including Defa5) are negatively correlated with the relative abundance of bacteria from the families *Lactobacillaceae* 10.1 and *Lactobacillaceae* FCS020 (Xia et al., 2022). Defensin levels are also negatively correlated with key obesity indicators, including body weight, body fat percentage, triglycerides, and total cholesterol levels (Xia et al., 2022). Correlation analysis suggests that electroacupuncture indirectly improves the gut microbiota of obese mice by promoting the production of intestinal defensins, thereby reducing obesity (Xia et al., 2022). Xia et al. demonstrated the anti-obesity mechanism of electroacupuncture by rescuing the gut microbiota and restoring damaged enteric neuronal systems (Si and Song, 2018; Dou et al., 2020). Recent studies have reported that electroacupuncture enhances the intestinal mucosal barrier, elevates secretory immunoglobulin A levels, and regulates the subtypes of T lymphocytes in the intestinal (Zhu et al., 2015; Zhang et al., 2018b). However, there is limited focus in the literature on elucidating the regulatory mechanisms of electroacupuncture from the perspective of mucosal innate immunity. The mechanisms underlying the anti-obesity effects of acupuncture treatment on the structure of the gut microbiota, whether these changes in the gut microbiota are causal risk factors for obesity or merely reflect acupuncture treatment, remain to be clarified. Changes in the gut microbiota may directly or indirectly target the brain via the VN stimulation or the neuroendocrine immune mechanism (El Aidy et al., 2015; Bliss and Whiteside, 2018). Current research indicates that acupuncture reduces food intake and body weight by regulating appetite-related hormones in the hypothalamus, including neuropeptide Y, cocaine- and amphetamine-regulated transcript, and proopiomelanocortin (Tian et al., 2005; Fei et al., 2011). Acupuncture can activate and release brain-gut peptides, which then mediate gastrointestinal motility and gut microbiota (Li et al., 2015). Further research is needed to reveal the precise mechanisms by which acupuncture regulates the gut microbiota to participate in the control of weight and lipid metabolism in abdominal obesity.

#### 2.3.2 Diabetes mellitus (DM)

DM is characterized by high blood sugar levels due to insulin deficiency. It is a set of metabolic disorders characterized by persistently high blood sugar levels. The development of DM is primarily caused by two factors: insufficient production of insulin by the pancreatic β cells and reduced responsiveness of the body to insulin (Pearson, 2019; Roden and Shulman, 2019). Type 2 DM (T2DM), the most common form of diabetes, is characterized by insulin resistance. Gut bacteria, also known as gut microbiota, play a role in the development and worsening of insulin resistance and T2DM (Tremaroli and Bäckhed, 2012). In individuals with abnormal metabolic states, an imbalance in the gut microbiota occurs, leading to the destruction of the integrity of the intestinal epithelial cells and increased leakiness of the intestinal mucosa. This, in turn, promotes the release of LPS and infiltration of inflammatory substances into the bloodstream, ultimately resulting in a mild inflammation (Liu et al., 2019a). Chronic low-level inflammation is a key factor contributing to insulin resistance (Almuraikhy et al., 2016). Some reports have suggested that the gut barrier is impaired and inflammation is higher in insulin-resistant mice, whereas electroacupuncture treatment can reduce the inflammatory state (Cani et al., 2009; An et al., 2022). By upregulating the expression of claudin-1, occludin, and ZO-1, electroacupuncture can enhance the integrity of the intestinal epithelial barrier (An et al., 2022). Additionally, electroacupuncture can reduce the production of substances that promote inflammation and increase the production of substances that counteract inflammation (An et al., 2022). Acupuncture can potentially influence the LPS levels and reduce the inflammatory response by modulating the composition of the gut microbiota, thus leading the treatment of T2DM. Animal experiments revealed a significant decrease in the abundance of *Firmicutes* and an increase in the abundance of *Bacteroidetes* in animal with T2DM compared with that in the normal controls. In patients with T2DM, there was a notable positive correlation between the proportion of *Bacteroidetes* to *Firmicutes* and blood glucose concentration (Larsen et al., 2010). Following electroacupuncture treatment (with a 3Hz expansion wave, current of 1mA), the abundance of *Firmicutes* increased, whereas that of *Bacteroides* decreased in patients with T2DM. Additionally, the proportion of *Firmicutes/Bacteroides* increased, leading to a decrease in LPS and TNF-α levels in the electroacupuncture group (Wang et al., 2022a). It is speculated that electroacupuncture has the potential to enhance overall inflammatory status and ultimately mitigate insulin resistance. This can be achieved through the regulation of gut microbiota, restoration of intestinal epithelial cell integrity, and reduction in intestinal mucosa permeability, thus hindering the entry of LPS into the systemic circulation. Furthermore, several studies have confirmed that electroacupuncture can restore the diversity of gut microbiota in mice with insulin resistance (Si et al., 2018; Wang et al., 2019). Electroacupuncture decreased the proportions of *Firmicutes* and specific bacteria such as *Odoribacter*, *Lachnospiraceae_UCG-006*, *Desulfovibrio*, *Oscillibacter*, and *Lachnoclostridium*, which are believed to be influenced by T2DM (An et al., 2022) (**Figure 2**). Furthermore, studies involving fecal microbiota transplantation have demonstrated that mice receiving microbiota from the electroacupuncture group exhibit improved blood glucose control and increased insulin sensitivity. This indicates that electroacupuncture can modulate gut microbiota and enhance glycometabolism (An et al., 2022). It is widely recognized that reducing systemic inflammation can facilitate the proper transmission of insulin, leading to decreased blood glucose levels. Studies have indicated that inflammatory elements can induce insulin resistance through the ikappaB kinase beta (IKKβ)/nuclear factor kappa B (NF-κB)-JNK- insulin receptor substrate-1 (IRS-1)- protein kinase B (AKT) signaling pathway present in the liver and muscles. Activation of the IKKβ/NF-κB-JNK-IRS-1 pathway by inflammatory cytokines can inhibit AKT phosphorylation, thereby reducing cellular glucose uptake and compromising insulin sensitivity (Park et al., 2016; Liu et al., 2019b; Sun et al., 2019). An et al. verified that electroacupuncture can boost insulin sensitivity by modulating the IKKβ/NF-κB-JNK-IRS-1-AKT signaling pathway in the liver and muscles (An et al., 2022). It is further speculated that electroacupuncture may enhance gut barrier function by regulating gut microbiota, thereby alleviating systemic inflammation and effectively reducing blood glucose levels by modulating the IKKβ/NF-κB-JNK-IRS-1-AKT signaling pathway.

#### 2.3.3 NAFLD

NAFLD poses a significant public health risk, with its global prevalence increasing at an annual rate of 25.2% (Asrani et al., 2019). NAFLD is closely associated with metabolic syndromes, including hypertension, hyperlipidemia, insulin resistance, and obesity (Ballestri et al., 2016). Further evidence suggests that the occurrence and progression of NAFLD are driven by an imbalance in the gut-liver axis and the metabolism of the gut microbiota (Sharpton et al., 2019). Within 14 days, the introduction of fecal samples from women with obesity and NAFLD into rats fed a regular diet significantly increased the liver triglyceride content (Hoyles et al., 2018). Le Roy et al. reported that when GF mice were transplanted with fecal samples from hyperglycemic mice and fed a HFD for 16 weeks, bullous steatosis was observed in their liver cells (Le Roy et al., 2013). Moreover, when mice with HFD-induced NAFLD were administered probiotics, such as *Pediococcus* and *Lactobacillus lactis*, for 8 weeks, there was an improvement in NAFLD symptoms (Yu et al., 2021). After 6 weeks of acupuncture, Wang et al. observed noticeable improvements in hepatic steatosis, lipid accumulation, inflammatory reactions, and deceleration in the progression of NAFLD. Meanwhile, the F/B ratio of the HFD mice decreased after acupuncture treatment (Wang et al., 2023). Clinical studies have found a positive correlation between the F/B ratio and steatosis in patients with NAFLD (Jasirwan et al., 2021). The F/B ratio increased in mice with HFD-induced NAFLD and decreased following treatment with bilberry anthocyanin (Nakano et al., 2020). Acupuncture also significantly elevated the levels of *Bacteroidales_S24–7_group*, *Blautia*, and *Prevotellaceae* (Wang et al., 2023) (**Figure 2**). *Bacteroidales_S24–7_group* bacteria can produce butyric acid, which may slow NAFLD progression (Endo et al., 2013; Zhou et al., 2017a). The *Blautia* genus is also acknowledged as a probiotic with the potential to improve metabolic disorders by generating butyric acid, thereby promoting gut health (Liu et al., 2021). Ozata et al. found a significant negative correlation between increased abundance of *Blautia* and visceral fat accumulation (Ozato et al., 2019). Although dietary fiber has been associated with improved glucose metabolism through an increase in *Prevotella* abundance, it is worth noting that *Prevotella* has also been linked to adverse physiological effects, such as insulin resistance (Pedersen et al., 2016). However, in a study by Wang et al., acupuncture significantly increased the abundance of *Prevotella_9* (Wang et al., 2023). Therefore, acupuncture may suppress hepatic inflammatory reactions and slow NAFLD progression by regulating the composition of certain gut microbiota. Serum lipid metabolism parameters, hepatic steatosis, and inflammatory factors were found to be closely related to the bacterial community in db-RDA analysis (Wang et al., 2023). The species in the acupuncture group were more like those in the control group than to those in the model group. This further indicates that acupuncture may regulate lipid metabolism, inflammatory response, and hepatic steatosis by influencing the gut microbiota.

### 2.4 Nervous system disease

#### 2.4.1 Parkinson’s disease (PD)

PD, a common neurodegenerative disease, is characterized by pathological features of dopamine fibers and neuronal loss in the striatum and substantia nigra. Furthermore, the central nervous system is affected by α-synuclein accumulation, which leads to increased neuroinflammation (Hartmann, 2004). Irregularities in gut microbiota play an important role in the pathogenesis and development of the gut-microbiota-brain axis imbalance in PD, leading to the generation of neuroinflammation and the deficiency of dopaminergic neurons (Sampson et al., 2016; Baizabal-Carvallo and Alonso-Juarez, 2020). Previous studies have shown that acupuncture has anti-neuroinflammatory, neuroprotective, and anti-apoptotic effects in PD mouse models (Park et al., 2003; Jeon et al., 2008; Kim et al., 2011a, 2011b). Genus-level analyses showed that the abundance of *Butyricimonas* and *Parabacteroides* decreased after acupuncture in a PD mouse model. Spearman’s correlation analysis revealed that the abundance of *Butyricimonas* was positively correlated with reduced anxiety and improved motor function (Jang et al., 2020). In previous experiments, it was observed that the GF mice, colonized with microbiota from patients with PD, exhibited a decrease in the abundance of *Butyricimonas* (Sampson et al., 2016). *Butyricimonas* is a butyrate producer with anti-inflammatory properties. The increase in *Butyricimonas* may play a crucial role in the anti-inflammatory effects observed during acupuncture therapy in mice with PD (Shiraï et al., 1988). After acupuncture-fecal microbiota transplantation, motor dysfunction in PD mice improved, indicating that acupuncture could improve PD symptoms through changes in the gut microbiota. In addition, the metabolic pathways involved in tetracycline biosynthesis, glutathione metabolism, and photosynthesis were significantly enriched, particularly in the PD pathway. Interestingly, this pathway was inactivated after acupuncture treatment (Jang et al., 2020). Another study reported that the abundance of *Erysipelotrichaceae* significantly decreased after electroacupuncture treatment (frequency of 2/100Hz) (Han et al., 2021a), whereas another *in vivo* experiment found that the abundance of *Erysipelotrichaceae* increased in PD mice (Lai et al., 2018). A recent review revealed that the abundance of *Erysipelotrichaceae* increased in patients with PD (Boertien et al., 2019). It has been reported that *Erysipelotrichaceae* may play a pivotal role in inflammation, which is positively correlated with IL-6, TNF-α, and CD14 levels (Dinh et al., 2015; Kaakoush, 2015; Li et al., 2019b). Acupuncture therapy reduces the activation of microglia and astrocytes, inhibits the increase in Bax expression, as well as levels of NF-κB and TNF-α. Acupuncture also exerts neuroprotective effects on dopaminergic neurons by suppressing the activation of neuroinflammation and apoptosis, improving motor dysfunction and associated anxiety in PD mice (Jang et al., 2020). While the exact mechanism of acupuncture in the gut-brain axis in PD is still unknown, the VN may play a key role in the connection between gut microbiota and brain induced by acupuncture. The communication between microbiota, gut, and brain through the microbiota-gut-brain axis is bidirectional, with the autonomic nervous system playing a crucial role (Borovikova et al., 2000; Bonaz et al., 2018). The VN is a major component of the parasympathetic nervous system, a mixed nerve with both afferent and efferent fibers. It can sense metabolites of gut microbiota and relay gut information to the central nervous system. This sensory information is integrated into the central autonomic network, which then generates responses through autonomic pathways (Bonaz et al., 2018). It is well known that acupuncture effects are related to the autonomic nervous system (Park and Namgung, 2018). These changes start from local responses to acupuncture. Acupuncture stimulation leads to the release of adenosine and histamine, as well as the expression of pERK and TRPV1 in the skin and muscle layers, activating neural transmission in the central nervous system (Goldman et al., 2010; Abraham et al., 2011; Park et al., 2014; Huang et al., 2018a). Signals transmitted to the brain impact various regions such as the prefrontal cortex, hypothalamus, and solitary nucleus, activating the central autonomic network to produce stimuli for the efferent VN (Borovikova et al., 2000; Chae et al., 2009; Cai et al., 2018). Studies suggest that the VN mediates acupuncture’s anti-inflammatory effects through the cholinergic anti-inflammatory pathway (Du et al., 2013). It is also believed that the activated efferent VN can mediate peripheral anti-inflammatory responses, thereby alleviating gut permeability and modulating changes in microbial composition (Borovikova et al., 2000; Zhou et al., 2013). Alterations in gut microbiota induced by acupuncture may contribute to anti-Parkinsonian effects in the brain, potentially through VN and/or humoral pathways. Further research is needed to elucidate the detailed mechanisms of acupuncture-induced gut-brain connections in PD models.

#### 2.4.2 Alzheimer’s disease (AD)

AD is a neurodegenerative condition characterized by cognitive impairments and memory decline, resulting in dementia (Scheltens et al., 2016). Experimental and clinical studies have consistently shown reduced gut microbiota diversity in both animal models and patients with AD. Furthermore, changes in the structure of the gut microbiota were observed (Leblhuber et al., 2021). Analysis of fecal samples from senescence-accelerated mouse prone 8 mice in animal experiments revealed a decrease in gut microbiota diversity, compared with senescence-accelerated prone 1 mice, with a significant reduction in *Lactobacillus* in the bacterial structure. *Bacteroides*, such as *Lachnospiraceae_NK4 A136* and *Alistites*, were significantly increased (Zhan et al., 2018; Sheehan and Musiek, 2020). Clinical data on the gut microbiota of older adults also confirmed the findings of the animal studies. The clinical results showed significant differences in the composition of the gut microbiota at the species and genus levels between patients with AD and healthy older adults, with a significant decrease in the abundance of *Firmicutes* and *Actinobacteria* (especially *Bifidobacterium*) and a significant increase in the abundance of *Pseudobacteria* and *Proteobacteria* (Askarova et al., 2020). Other study has also validated that acupuncture treatment can effectively regulate the dysbiosis of gut microbiota in amyloid precursor protein/presenilin 1(APP/PS1) mice by promoting the increase of *Bacteroidetes* and suppressing the decrease of *Proteobacteria* and *Escherichia-Shigella* (Hao et al., 2022) (**Figure 2**). Research indicates that probiotics, such as *Bifidobacterium bifidum*, *Lactobacillus acidophilus*, and *Bifidobacterium lactis*, can protect the brain by improving the intestinal barrier and BBB integrity, thereby reducing inflammation (Akbari et al., 2016; Plaza-Díaz et al., 2017). An animal study showed that electroacupuncture with continuous wave frequency of 50Hz and the intensity of 1mA improved spatial learning and memory impairments in AD mice, reduced serum LPS levels, restored BBB function, increased the abundance of *Lactobacillus* and *Bifidobacterium*, and reduced the abundance of *E. coli* and *B._fragilis* (He et al., 2021). Furthermore, electroacupuncture inhibited the TLR4/NF-κB pathway by suppressing LPS levels, which could activate the pathway to produce the pro-inflammatory cytokines and cause neurodegeneration (He et al., 2021). These results indicate that electroacupuncture therapy can improve cognitive function by modulating the balance of the gut microbiota. This study suggests that an imbalance in the gut microbiota may trigger inflammation in the brain, and electroacupuncture therapy can safeguard the central nervous system by suppressing the TLR4/NF-κB signaling pathway (He et al., 2021).

#### 2.4.3 Spinal cord injury (SCI)

SCI is a neurological condition caused by the impairment of the spinal cord, resulting in various functional abnormalities (McDonald and Sadowsky, 2002). Increasing evidence suggests that dysregulation of the gut microbiota plays a crucial role in the development and manifestation of the pathological mechanisms and clinical symptoms of SCI (Gungor et al., 2016). Normally, *Proteobacteria* constitute only a minor fraction of the gut microbiota under physiological conditions (Shin et al., 2015). Significant changes have been observed in the composition and evenness of the gut microbiota in rats after SCI. At the phylum level, the abundance of *Proteobacteria* in the SCI group significantly increased but significantly decreased after electroacupuncture treatment and dilatational wave was 2/15Hz, current intensity was 1–2mA (Cheng et al., 2022). Abnormalities in the gut immune system may lead to short-term expansion of *Proteobacteria*-dominated communities, resulting in gut inflammation and sensorimotor dysfunction (Maharshak et al., 2013). Owing to their strong adaptability in SCI rats, *Proteobacteria* expand into dominant microbial communities and exacerbate disease progression. However, in rats, electroacupuncture inhibited the expansion of *Proteobacteria* (Cheng et al., 2022). In the process of improving SCI through microbial community modulation by electroacupuncture, communication between the microbial community and SCI recovery is important and may depend on various pathways, including the immune system, gut microbial metabolites, and tryptophan metabolism system, which may involve metabolites, such as catechin (Schroeder and Bäckhed, 2016; Sharon et al., 2016). Catechins are known for their antioxidant, anti-apoptotic, and anti-inflammatory properties (Khalatbary and Khademi, 2020). Kutschera et al. demonstrated the involvement of *Clostridium orbiscindens* and *Eggerthella lenta*, which were extracted from the human gut, in catechins metabolism (Kutschera et al., 2011). The research conducted by Cheng et al. also found differences in the enrichment levels of *Clostridia* in different groups. Specifically, the abundance of *Clostridia* significantly increased in the SCI group, but decreased significantly after electroacupuncture treatment, with no change observed after faecal microbiota transplantation (Cheng et al., 2022). Integrated analysis of the microbiota and metabolome revealed significant correlations between the catechin precursor pyrocatechol and various microbiota in the class *Clostridia*, including *Anaerofustis*, *Subdoligranulum* and *Faecalibacterium*. Therefore, catechins may improve SCI through electroacupuncture treatment by stimulating changes in the microbial metabolite levels. Moreover, studies have shown that electroacupuncture treatment can improve gut function and restore 5-HT expression in SCI rats, indicating modulation of the 5-HT system by electroacupuncture during SCI treatment (Cheng et al., 2022). Nevertheless, the specific mechanism underlying the modulation of 5-HT by the microbial communities, either independently or synergistically with metabolites, require further investigation.

#### 2.4.4 Post-operative delirium

Post-operative delirium is a common neurocognitive disorder during the perioperative period (Evered et al., 2018). Clinical studies have shown that more than 80% of patients experience post-operative pain, which is closely linked to delirium (Apfelbaum et al., 2003; Avidan et al., 2017; Li et al., 2021c). Previous studies have indicated a relationship between gut microbiota dysbiosis and pain (Shen et al., 2017b; Lin et al., 2020a). A survey report suggested that anesthesia or surgery increased the abundance of *Lactobacillus* and delirium-like behavior in mice (Liufu et al., 2020). Gut microbiota imbalance enhances BBB permeability by regulating the production of SCFAs (Braniste et al., 2014). Increased BBB permeability may worsen neuroinflammation and aggravate the neuropathological mechanisms of delirium (Kowalski and Mulak, 2019; Zhu et al., 2020). According to a study by Yang et al., pain from surgery leads to delirium-like symptoms in mice, involving the activation of microglial cells and changes in dendritic spines influenced by the gut microbiota (Yang et al., 2022a). Postoperative pain increased the relative abundance of *Verrucomicrobia*, *Cyanobacteria*, *Lactobacillaceae*, and *Ak*kermansia in mice. Applying electroacupuncture (dense-disperse wave forms 100/2Hz) to specific acupoints, such as DU20 + Yongquan (KI1) or Huantiao (GB30) + ST36, can prevent or alleviate delirium-like symptoms caused by surgical pain, while also modulating the gut microbiota, microglial cells, and dendritic spine function (Yang et al., 2022a). Stimulation with electroacupuncture at DU20 and KI1 inhibited the activation of brain microglial cells (Tang et al., 2016). The use of electroacupuncture showed protective effects on the brain under CUMS conditions. This was achieved by boosting the production of BDNF and improving hippocampus neurogenesis of a depression rat model (Mao et al., 2020). Moreover, applying electroacupuncture stimulation at DU20 and KI1 not only ameliorated cognitive deficits but also enhanced the adaptability of synapses by activating the protein kinase A (PKA)/cyclic adenosine monophosphate response element-binding protein (CREB) signaling pathway (Zheng et al., 2016a). Separate research indicated that electroacupuncture treatment at KI1 effectively prevented spatial memory impairments in rodents by modulating nicotinamide adenine dinucleotide phosphate oxidase 2 (NOX2) activity in the hippocampus (Wu et al., 2017). NOX2 acts as a key regulator of oxidative stress and neuroinflammation. In a mouse model of PD, electroacupuncture therapy alleviated behavioral changes by adjusting the gut microbiota and suppressing neuroinflammation (Han et al., 2021b).

### 2.5 Cardiovascular disease

#### 2.5.1 Myocardial infarction (MI)

MI is a term used to refer to an event of heart attack. The main symptoms of MI include chest discomfort that may extend to the left arm or neck, breathlessness, vomiting, irregular heartbeat, queasiness, perspiration, uneasiness, and weariness (Valensi et al., 2011). Although the survival rate and clinical outcomes after MI have improved through the restoration of blood flow, there are still many complications, such as adverse left ventricular remodeling, chronic heart failure, and various pathological changes in the myocardium, which are collectively referred to as myocardial ischemia-reperfusion injury (MIRI) (Lesnefsky et al., 2017). A previous study showed that decreased cardiac function could lead to reduced intestinal blood flow, induce intestinal morphological alterations, and cause changes in the gut microbiota composition (Sandek et al., 2014). Changes in gut microbiota composition can reduce the risk of cardiovascular diseases (Battson et al., 2019). Bai et al. found that electroacupuncture at Neiguan (PC6) altered the composition of the gut microbiota. After 7 days of electroacupuncture treatment and stimulated with an intensity of 2mA and a frequency of 2/15Hz, the relative abundances of *RF39*, *S24–7*, *Desulfovibrio* and *Allobaculum* were significantly reduced compared with those in MIRI rats (**Figure 2**), which were closely related to LPS production and the gut barrier (Bai et al., 2021). *Desufovibrio* has been proven to interact with intestinal epithelial cells and trigger epithelial cell apoptosis (Coutinho et al., 2017). RF39 expression was positively correlated with increased inflammatory cytokine levels (Zhang et al., 2019b). Interestingly, *Allobaculum* can produce SCFAs (Zhang et al., 2019b), which enhance intestinal barrier function, inhibit pathogenic bacterial growth, reduce inflammation, and modulate immune responses (Kelly et al., 2015; Asarat et al., 2016; Tang et al., 2019a). However, in an *in vivo* experiment conducted by Bai et al (Bai et al., 2021), it was found that the level of *Allobaculum* increased further with electroacupuncture intervention after MIRI. Considering that electroacupuncture treatment improved intestinal barrier function and reduced inflammation and pathogenic bacteria (Bai et al., 2021), it is speculated that SCFAs may be involved in the protective effects of electroacupuncture in improving intestinal barrier function and reducing inflammation. However, *Allobaculum* may not be the primary producer of SCFAs after electroacupuncture intervention. Another hypothesis was that SCFAs stimulate lipid oxidation (Hosseini et al., 2011). Previous studies have shown that fatty acid oxidation is inhibited after myocardial ischemia (Bai et al., 2020), and electroacupuncture intervention reduces SCFAs production through *Allobaculum* and modulates lipid metabolism, whereas electroacupuncture improves intestinal barrier function and reduces inflammation through mechanisms other than SCFAs. However, there is currently a lack of in-depth research, and further exploration is needed to investigate the relationship between electroacupuncture intervention after MIRI and SCFAs production, as well as the pathways through which electroacupuncture affects PC6 to modulate the potential biological functions of the gut microbiota. Additionally, the crucial role of these microbiota in electroacupuncture-induced myocardial protection needs to be examined.

#### 2.5.2 Ischemic stroke

Ischemic stroke, characterized by its high incidence, has a major influence on well-being and carries a higher mortality risk, posing a serious medical problem worldwide (Krishnamurthi et al., 2020). Gut microbiota has been identified as a novel target for stroke treatment. Previous studies have revealed a reciprocal and dynamic link between the gastrointestinal system, its microorganisms, and the brain (Durgan et al., 2019). Stroke can induce dysbiosis of the gut microbiota, manifesting as diminished diversity, depletion of probiotics, promotion of pathogenic or opportunistic bacterial populations, and even their migration and dispersal to other organs, thereby giving rise to additional complications, such as aggravated systemic inflammation and stroke severity (Stanley et al., 2016; Wu et al., 2021b; Xu et al., 2021a; Zhu et al., 2021a). Acupuncture, a special traditional Chinese medicine therapy, has been used for thousands of years to treat strokes in China. Several meta-analyses have shown that acupuncture can effectively ameliorate sequelae, such as cognitive impairment, dysphagia, and hemiplegia (Long and Wu, 2012; Liu et al., 2014; Fan et al., 2020). Furthermore, acupuncture can treat stroke by modulating gut microbiota (Xian et al., 2022). In a study by Xian et al., compared with a model group, an acupuncture group showed an increased relative abundance of the phylum *Firmicutes*, as well as the genera *Actinobacteriota*, *Lactobacillus*, *Turicibacter*, and *Bifidobacterium*. Compared with the model group, the relative abundance of *Escherichia-Shigella* decreased in the acupuncture group (Xian et al., 2022). Many beneficial bacteria belonging to the *Firmicutes* family, including *Lactobacillus* and specific bacteria that produce butyrate, are important participants in the production of SCFAs (Mrozinska et al., 2016; Thomas et al., 2020). Chen et al. demonstrated that transplantation of gut microbiota which produce increased levels of SCFAs and butyrate could serve as a therapeutic approach for ischemic stroke (Chen et al., 2019). *Bifidobacterium* is the prevailing bacterium of the phylum *Actinobacteria* residing in the human gut and plays a pivotal role in fortifying the intestinal barrier, thereby averting the entry of noxious agents, such as LPS, into the bloodstream (Binda et al., 2018). *Escherichia-Shaigella*, a member of the phylum *Proteobacteria*, consists of pathogenic bacteria that produce a multitude of LPS (Lin et al., 2020b). Studies have indicated that increased levels of LPS can worsen stroke outcomes by directly binding to TLR4 and activating NF-κB, triggering the production of pro-inflammatory cytokines (Hakoupian et al., 2021). The mechanisms of acupuncture in stroke treatment may be related to the maintenance of gut microbiota homeostasis and regulation of metabolic dysfunction, which could be an effective therapeutic strategy for stroke. However, the relationship between gut microbiota and acupuncture, as well as the gut microbiota and metabolites, remains unclear. Further studies involving GF rats, fecal microbiota transplantation, and metagenomics are necessary to elucidate the underlying mechanisms of acupuncture in stroke treatment. According to previous reports, immune signals can be exchanged between the brain and gut following gut microbial dysbiosis after a stroke (Yang et al., 2022c; Zhao et al., 2022). After cerebral ischemia, the gut microbiota further impairs the immune system, modulates lymphocytes, affects IL-17 secretion, affects Tregs and γδT cells, and exacerbates gut and brain damage (Singh et al., 2016; Wang et al., 2022b). Treg cells suppress postischemic inflammation by secreting IL-10, which plays a neuroprotective role (Liesz et al., 2015). IL-17 knockdown alleviates brain injury and restores gut microbiota homeostasis (Cheng et al., 2019; Dai et al., 2019). The transplantation of a healthy gut microbiota and administration of external substances, such as SCFAs and fructose, triggers the transformation of T cells into Th1 cells and promotes the release of IL-10 to suppress inflammation (Sun et al., 2018; Zeng et al., 2019; Sadler et al., 2020). Dysbiosis of the gut microbiota after cerebral ischemia leads to inflammation, and increased expression of inflammatory cytokines further exacerbates dysbiosis (Zhang et al., 2023). Zhang et al. confirmed that electroacupuncture (2Hz frequency with continuous waves) regulates IL-17 and IL-10 levels in the brain and colon tissues through the brain-gut axis, significantly improves neurological function, neuronal and intestinal damage, and modulates the differential distribution of microbial communities in the mouse intestine after cerebral ischemia-reperfusion (Zhang et al., 2023). Proteobacterial levels were significantly elevated in the middle cerebral artery occlusion group after cerebral ischemia and were reduced by electroacupuncture treatment. The levels of beneficial bacteria, such as *Firmicutes* and *Verrucomicrobia*, can be increased by electroacupuncture. Furthermore, electroacupuncture increases the levels of *Akkermansia*, which safeguards the integrity of gut epithelial cells and the protective mucus layer, serving a protective function in metabolism (Zhang et al., 2023). *Akkermansia* is linked to cardiovascular and cerebrovascular diseases, after cerebral ischemia, it promotes metabolic balance owing to reduced blood flow.

#### 2.5.3 Hypertension

Hypertension is the primary cause of cardiovascular diseases in China (Wang et al., 2018a; Wang et al., 2018b). Electroacupuncture has been shown to effectively lower both systolic and diastolic blood pressure in individuals with hypertension. Electroacupuncture with 2Hz continuous wave and intensity varied from 0.1 to 2.0mA also significantly reduced the abundance of Firmicutes and the F/B ratio (**Figure 1**) (Wang et al., 2021a). An increase in the F/B ratio was observed in various animal models of hypertension (Yang et al., 2015; Adnan et al., 2017). After electroacupuncture therapy, the relative abundance of *Escherichia-Shigella* decreased, whereas that of *Blautia* increased. Furthermore, *Escherichia-Shigella* was positively correlated with diastolic blood pressure, whereas *Blautia* was negatively correlated with systolic blood pressure and diastolic blood pressure (Wang et al., 2021a). *Escherichia-Shigella* is a group of Gram-negative bacteria with LPS in their cell walls (Sircana et al., 2019). LPS activates the TLR4 pathway, inducing the activation of pro-inflammatory cytokines (TNF-α, IL-6, and IL-1), leading to inflammation (Lamping et al., 1998; Beutler, 2002; Yin et al., 2013). Bomfim et al. described that chronic treatment with anti-TLR4 antibodies inhibited TLR4 activation, reduced blood pressure, and endothelial dysfunction in spontaneously hypertensive rats (Bomfim et al., 2012). The decrease in Escherichia-Shigella levels after electric acupuncture treatment may indicate an improvement in the host’s inflammatory status. A recent review summarized the mechanisms of acupuncture in treating hypertension, showing that the renin-angiotensin-aldosterone system, vascular endothelium, oxidative stress response, and neuroendocrine system are all involved in the anti-hypertensive effects of acupuncture, which are influenced by the gut microbiota (Li et al., 2019a). It is difficult to say which of these two aspects (plant group and host) is the direct target of acupuncture. Therefore, further research is needed to determine the contribution of specific bacteria controlled by electric acupuncture to blood pressure regulation.

### 2.6 Other diseases

#### 2.6.1 Cancer

Cancer is a significant global public health challenge and is the leading cause of mortality in both more and less economically developed countries (Chen et al., 2016). The development of tumors is related to the body’s (Wu et al., 2021a). The primary defense mechanism of the body, cellular immunity plays a crucial role in responding to internal threats. T cells and natural killer (NK) cells actively participate in the immune process. When the body has a tumor, the imbalance in the proportion of immune cells and related cytokines leads to the destruction of the immune system (Vivier et al., 2008; McKenzie and Valitutti, 2023). Through acupuncture, the levels of CD3+ and CD4+ T cells can be elevated while reducing the presence of cytotoxic CD8+ T cells. This rebalancing effect helps normalize the CD4+/CD8+ ratio, ultimately enhancing the body’s immune capabilities (Wu, 1995). Acupuncture can also increase the expression of IFN-γ and IL-6 in the serum of osteosarcoma cell mice (Xu et al., 2020). The combination of cisplatin and acupuncture can significantly reduce the levels of TNF-α in late-stage lung cancer patients, potentially improving the patients’ immune function and clinical efficacy (Zhang et al., 2015b). Xu et al. also found that acupuncture can slow down tumor growth and metastasis by reducing the secretion of NK cells. Furthermore, acupuncture intervention delayed the changes in immune cells in mice with tumor burden, with higher expression of helper T cells and lower expression of cytotoxic T cells in the acupuncture group compared to the model group (Xu et al., 2020). Once again, it confirms that acupuncture affects the development of tumors by regulating immune responses. Current researches have demonstrated differences in the composition of gut microorganisms between patients with cancer and healthy individuals. The occurrence of cancer often coincides with or leads to alterations in gut microbiota (Huang et al., 2018b; Sims et al., 2019; Zheng et al., 2019). Acupuncture regulated the expression of specific bacteria, and the relative abundances of *Bacteroidetes* and *Firmicutes* in the acupuncture group were closer to those in the control group and different from those in the model group (Xu et al., 2020). Xu et al. also found a decrease in the relative abundance of *Aestuarispira*, *Alloprevotella*, *Parasutterella*, *Eubacterium*, *Prevotella*, *Streptoccus*, *Bacteroides*, *Murimonas*, and *Parabacteroides* in osteosarcoma mice, and acupuncture treatment regulated the changes in these genera (Xu et al., 2020). It is worth noting that cancer patients are often treated with high-dose chemotherapy, which typically results in strong gastrointestinal reactions. Various treatments for other cancers often lead to diarrhea as well (Redman et al., 2014). These reports suggest that cancer is related to disturbances in gut microbiota, which is the target of current Western medical treatment. The above studies suggest that acupuncture has the potential to support the body’s immune system, control the release of inflammation-inducing substances, balance the gut bacteria, and disrupt the growth of tumors. Acupuncture, when used in conjunction with other treatments and approaches, shows promising potential in enhancing cancer care and control.

#### 2.6.2 Cancer-related fatigue

Cancer-related fatigue is the most prevalent adverse effect of breast cancer chemotherapy. It is characterized by constant exhaustion and a decline in mood and cognitive function that cannot be alleviated by rest and sleep, inducing decreased physical function and overall quality of life (Mohandas et al., 2017; Xiao et al., 2021). Research indicates that the gut microbiota, metabolic system, autonomic nervous system, ENS and central nervous system form a complex signaling network known as the gut microbiota-gut-brain axis (Quigley, 2017). The gut microbiota-gut-brain axis is closely associated with cancer-related fatigue. Mounting evidence suggests that an imbalance in the gut microbiota can disturb the permeability of the intestinal barrier, leading to intestinal inflammation, peripheral blood inflammation response, and, ultimately, BBB dysfunction. This disruption triggers an inflammatory response in the central nervous system, causing functional impairment of the hypothalamic-pituitary-adrenal (HPA) axis and resulting in neurological disorders (Iannone et al., 2019; Agirman et al., 2021). A central inflammatory response may lead to the expression of HPA axis-related factors, such as corticotropin-releasing hormone and cortisol, and disruption of adrenocorticotropic hormone, exacerbating fatigue symptoms (Tell et al., 2014). Acupuncture therapy has been found to have anti-fatigue effects and can modulate gut microbiota dysbiosis in fatigued mice (Lv et al., 2022). Acupuncture can modulate various aspects of the gut-brain axis and restore homeostasis of the gut microbiota (Jang et al., 2020). Studies have confirmed that the fatigue level of patients with breast cancer in the recovery period was notably lower after acupuncture at Qihai (CV6), Sanyinjiao (SP6), CV4 and ST36 than that in the sham acupuncture group, with a significant improvement in quality of life (Zhang et al., 2018a; Jang et al., 2020; Li et al., 2020). Animal experiments found that at the phylum level, the abundance of *Bacteroidetes* in mice with cancer-related fatigue significantly decreased after breast cancer chemotherapy, whereas the abundance of *Proteobacteria* increased. However, after acupuncture, the abundance of *Bacteroidetes* and *Firmicutes* increased, whereas that of *Proteobacteria* decreased (Lv et al., 2022). Additional data confirmed that *Firmicutes* and *Bacteroidetes* enhanced ginsenosides metabolism, reduced inflammation, and indirectly ameliorated fatigue (Zhou et al., 2016). At the genus level, the abundance of *Lactobacillus* decreased and that of *Burkholderia-Caballeronia-Paraburkholderia* increased in mice with cancer-related fatigue after breast cancer chemotherapy. However, after acupuncture therapy, the abundance of *Lactobacillus*, *Streptococcus*, and *Burkholderia-Caballeronia-Paraburkholderia* reversed (Lv et al., 2022). Recent research indicates that the use of *Lactobacillus*, a probiotics, can effectively prevent undesirable side effects such as heart toxicity, intestinal harm, tiredness, and disruptions to sleep patterns caused by the medication used in chemotherapy treatments (Zhao et al., 2018). A study conducted by Zulpa et al. discovered that the presence of *Burkholderia* may trigger an inflammation-based response, leading to a range of negative effects in individuals undergoing chemotherapy treatment (Zulpa et al., 2021). Acupuncture therapy also reduces the inflammatory response caused by cancer-related fatigue and restores the levels of tight junction proteins in the intestinal barrier. Furthermore, studies have demonstrated that the inflammatory response in the central nervous system can disrupt the function of the HPA axis, leading to a decrease in cortisol synthesis and release, ultimately resulting in cancer-related fatigue. The HPA axis includes cortisol, corticotropin-releasing hormone, and adrenocorticotropic hormone (Saligan et al., 2015; Han et al., 2021a). Lv et al. found that after breast cancer chemotherapy, cancer-related fatigue notably reduced the levels of corticotropin-releasing hormone and cortisol and increased the level of adrenocorticotropic hormone in the serum, which was also restored by acupuncture therapy (Lv et al., 2022). It is speculated that acupuncture can improve HPA axis dysfunction by influencing the gut microbiota-gut-brain axis pathway, potentially alleviating fatigue symptoms.

#### 2.6.3 Sleep disturbances on IBD

The impact of sleep disturbances on IBD has recently gained substantial attention, with an increasing number of studies exploring the interconnectedness between these two conditions (Ali and Orr, 2014). Research has shown that patients with IBD often experience restless sleep and severe sleep disturbances, even outside the active phase of the disease (Ranjbaran et al., 2007). Sleep disruptions are linked to IBD disease activity, triggering inflammation in the intestines (Ali et al., 2013). The gut microbiota can be viewed as an internal ecosystem in mammals that sustains and interacts with the body. In recent years, it has been discovered that chronic inflammation-related diseases, such as IBD, are closely associated with an imbalance or disruption of the normal microbial community or circadian rhythm (Voigt et al., 2014). Furthermore, the well-being of the intestinal lining relies greatly relies on the ability of intestinal epithelial cells to detect and respond to changes in the microbial composition (Mukherji et al., 2013). However, the composition and physiological oscillations exhibited by the gut microbiota also follow the feeding rhythms of the host and change periodically (Thaiss et al., 2014). Recent studies have indicated that certain strains of gut bacteria directly influence IBD and sleep fragmentation (Ohkusa and Koido, 2015; Sanford et al., 2021). Animal experiments have shown that electroacupuncture treatment (dilatational wave with frequency of 12Hz) can effectively reduce intestinal inflammation and influence gut microbiota in UC mice with sleep fragmentation (Liu et al., 2022). Another study demonstrated that mice treated with electroacupuncture for sleep fragmentation-induced UC not only alleviated the symptoms and signs of UC but also restored some of the gut microbiota (Wei et al., 2019). For example, the levels of *Lactobacillus* spp. and *Lachnospiraceae* bacteria increased, whereas those of *Clostridium bifermentans* decreased (Wei et al., 2019). Baldelli et al. confirmed that electroacupuncture effectively regulates the presence of *Enterobacteriaceae*, a known contributor to the development and advancement of IBD (Baldelli et al., 2021). In addition, electroacupuncture reduces the level of IL-6 in the plasma, which affects sleep, and restores the abundance and diversity of the microbiomes (Wei et al., 2019). Although human studies have shown that IL-6 may improve sleep efficiency, its impact on waking after sleep onset remains relatively minor (Smith et al., 2019). Recent research on sleep deprivation has confirmed that both sleep efficiency and total sleep time are positively associated with the overall diversity of the microbiome, whereas waking after sleep onset shows a negative correlation (Lee-Chiong, 2005). Therefore, it is speculated that electroacupuncture may improve sleep and alleviate the symptoms of mice with UC by modulating the gut microbiota.

#### 2.6.4 Osteoarthritis (OA)

OA is a prevalent chronic joint disease that manifests as a gradual deterioration of joint cartilage, formation of osteophytes, and inflammation of the synovium (Sharma et al., 2000). Recent discoveries have indicated that the gut microbiota could be a potential link between metabolism-induced OA and the activation of innate immune responses, which ultimately triggers systemic inflammation (Metcalfe et al., 2012). In an obesity-induced OA mouse model, it was found that electroacupuncture with an intensity of 2–3mA and a frequency of 30Hz at different acupoints caused different changes in the gut microbiota of mice (Xie et al., 2020). The relative abundance of *Clostridium*, *Akkermansia*, *Butyricimonas* and *Lactococcus* recovered after 2-week electroacupuncture treatment with three patterns at ST36, Yanglingquan (GB34), and ST36+GB34. The relative abundances of *Lactobacillus*, *Treponema*, *Roseburia*, and *Coprococcus* were increased by electroacupuncture at ST36+GB34, however, electroacupuncture at GB34 only increased the relative abundance of *Lactobacillus* and *Treponema*. Furthermore, the relative abundance of *Epulopiscium* was decreased by electroacupuncture at GB34 but increased by electroacupuncture at ST36, whereas there was no difference with electroacupuncture at ST36+GB34. Simultaneously, the three patterns of electroacupuncture improved matrix arrangement, cartilage lesion inhibition, tide line maintenance, and increased the expression of matrix metalloproteinase (MMP)-1 and MMP-13, which proved that electroacupuncture prevented cartilage loss in obesity-induced OA mice. Electroacupuncture administered at acupoints ST36 and GB34 reduced synovial fluid LPS levels and suppressed p65 and P-p65 (Xie et al., 2020). LPS plays a crucial role in activating the TLR4/NF-κB signaling pathway, which leads to inflammatory responses in obesity-related metabolic diseases (Cani et al., 2007). The effect of electroacupuncture at ST36+GB34 on suppressing the TLR4/NF-κB signaling pathway by reducing LPS levels, as well as the different changes in gut microbiota induced by the three electroacupuncture modes, still need to be further explored. Additionally, in individuals with knee OA (KOA), there was an increase in the abundance of *Streptococcus* (**Figure 1**) (Wang et al., 2021b). Research has shown that the abundance of *Streptococcus* is significantly correlated with the severity of knee joint effusion (Collins et al., 2015). After electroacupuncture treatment (density wave with frequency of 2/100Hz), the abundance of *Streptococcus* was reversed in patients with KOA (Wang et al., 2021b) (**Figure 2**). It has been hypothesized that electroacupuncture can effectively alleviate the manifestations and symptoms of KOA by inducing alterations in the composition of the gut microbial community, particularly by diminishing the level of *Streptococcus*. Additionally, Wang et al. observed a decrease in the abundance of *Bacteroides* and *Agathobacter* in patients with KOA (**Figure 2**). *Bacteroides* and *Agathobacter* were negatively correlated with the Western Ontario and McMaster Universities Osteoarthritis Index (WOMAC) total score as well as WOMAC stiffness, function, and pain scores (Wang et al., 2021b). It is well known that species in *Bacteroides* and *Agathobacter* species produce SCFAs through the fermentation of dietary fiber (Zhang et al., 2015a; Koh et al., 2016; Hua et al., 2020), which have beneficial effects on health owing to their anti-inflammatory properties (Kelly et al., 2015). Elevated concentrations of SCFAs in fecal matter can enhance energy acquisition from dietary fiber, inhibit the growth of harmful pathogens, and afford protection against inflammatory and colonic disorders (De Filippo et al., 2010). Wang et al. reported a substantial increase in the abundance of *Agathobacter* following electroacupuncture treatment, which coincided with pain relief (Wang et al., 2021b). Therefore, electroacupuncture treatment may achieve pain relief by increasing the abundance of beneficial bacteria, such as *Bacteroides* and *Agathobacter*, and by protecting the host from inflammation. Although there is currently no direct evidence to suggest that microbiota altered by electroacupuncture can relieve KOA pain, existing clinical studies provide indirect evidence that the gut microbiota may be involved in treatment.

#### 2.6.5 Stress urinary incontinence (SUI)

Middle-aged and older women are often affected by SUI. Involuntary urine leakage occurs when the abdominal pressure increases (D'Ancona et al., 2019). Studies conducted in both animals and humans have indicated the concurrent occurrence of lower urinary tract and intestinal disorders (Panicker et al., 2019). A healthy gut microbiome plays a crucial role in various bodily functions, including nutrient metabolism and intestinal mucosal growth and is closely linked to individual well-being (Jandhyala et al., 2015). In recent years, researchers have found that electroacupuncture stimulation of the lumbosacral region reduces the frequency and extent of urine leakage in women with SUI and that electroacupuncture stimulation also alters the species composition of the gut microbiota in SUI rats (Liu et al., 2017; Li et al., 2022a). By impeding collagen degradation in the anterior vaginal wall of the pelvic floor support tissue, electroacupuncture has the potential to reduce SUI in rats (Li et al., 2022b). After electroacupuncture treatment (density wave, 4/20Hz), SUI rats exhibited an increased abundance of *Blautia*, which was positively correlated with changes in leak point pressure within the electroacupuncture group (Li et al., 2022b). Nonetheless, further investigations are required to ascertain whether the influence of electroacupuncture on the gut microbial community in SUI rats is associated with a reduction in collagen degradation.

#### 2.6.6 Premature ovarian failure (POF)

POF is characterized by excessive loss of ovarian oocytes and abnormal sex hormone levels (Touraine, 2020). Recent research indicates that eating habits and the environment are closely linked to the development of POF, and an unhealthy diet high in fat and sugar can impair the ovarian function and ovum quality (Liu et al., 2005; Luisi et al., 2015; Wu et al., 2015; Szeliga et al., 2021). Previous studies have demonstrated that an unhealthy diet high in fat and sugar may disrupt the balance of microorganisms in the gut, thereby affecting the overall health of individuals (Choi et al., 2021; Tan et al., 2022). Several studies have suggested that through the gut microbiota, an unhealthy diet high in fat and sugar affects the communication between the gut and brain, playing a crucial role in modulating the production of gonadal hormones (Jašarević et al., 2016). Tan et al. discovered changes in ovarian function in mice receiving a fecal microbiota transplant from women with polycystic ovarian syndrome (PCOS) (Tan et al., 2022). Electroacupuncture can prevent follicle loss, increase the levels of anti-Müllerian hormones in the blood, and enhance antioxidant and anti-apoptotic activation (Zhang et al., 2018; Wang et al., 2019b; Zhang et al., 2019a). Additionally, electroacupuncture can ameliorate reproductive function by remodeling and adjusting the abundance of the gut microbiota (Zhang et al., 2022a). Meanwhile, diet high in fat and sugar can induce oxidative stress (Zhu et al., 2021b). Zhang et al. demonstrated that electroacupuncture modulates metabolic disorders and enhances reproductive function in a rat model with PCOS-like symptoms by modulating the gut microbiota (Zhang et al., 2022a). Geng et al. found that at the genus level, electroacupuncture significantly reduced the abundance of *Anaeroplasma*, *Alistipes*, *Pantoea*, *Rikenella*, *Anaerotruncus*, *Clostridium_sensu_stricto_1*, *Rikenellaceae_RC9_gut_group*, and *Ruminococcaceae_UCG-009* (Geng et al., 2022) (**Figure 2**). These microbial taxa were negatively correlated with the improvement of oxidative stress damage and the reduction of Fe^2+^ levels during electroacupuncture treatment, suggesting that electroacupuncture can suppress oxidative stress by modulating the gut microbiota (Geng et al., 2022). In the study by He et al., it was found that *Lachnospiraceae*, *Eubacterium coprostanoligenes*, and *Blautia* were dominant microbial groups in the POF group (He et al., 2022). Dysregulation of *Lachnospiraceae* can disrupt the glucose metabolism and promote inflammation (Vacca et al., 2020). After electroacupuncture treatment, *Anaeroplasma*, *Ruminococcaceae*, and *Eubacterium ventriosum* became the three most relatively expressed microbial groups (He et al., 2022). *Anaeroplasma* is an anti-inflammatory agent that contribute to maintain the balance of the intestinal immune system (He et al., 2019). Equally, *Ruminococcaceae* and *Eubacterium ventriosum*, both common beneficial bacteria in the intestinal, exhibit anti-inflammatory functions (Shang et al., 2016; Wei et al., 2018). As member of SCFAs, *Ruminococcaceae* is believed to help maintain intestinal immune homeostasis. A cross-sectional study reported that *Ruminococcaceae* can increase estrogen levels (Flores et al., 2012), confirming that electroacupuncture can increase the abundance and diversity of probiotics in the intestines of POF mice. He et al. also found that electroacupuncture can restore serum levels of follicle-stimulating hormone (FSH), luteinizing hormone, estradiol (E2), and anti-Mullerian hormone in POF mice, activate the phosphatidylinositol 3-kinase (PI3K)-AKT signaling pathway to promote ovarian cell proliferation (He et al., 2022). The PI3K-AKT signaling pathway plays a vital role in supporting oocyte growth, initial follicle formation, and the multiplication of granulosa cells (Zhou et al., 2017b). When FSH links up with receptors on the granulosa cell surface, it triggers the initiation of the PI3K-AKT pathway, which in turn facilitates the maturation of granulosa cells (Shen et al., 2017a). Moreover, E2 engages with estrogen receptors-alpha (ER-α) and estrogen receptors-beta (ER-β) within the ovary (Tang et al., 2019b). ER-α specifically binds with the p85 subunit of PI3K to kickstart the AKT activity (Dornan et al., 2020; Wang et al., 2020b). Subsequently, AKT activation prompts mTOR activation, ultimately promoting the proliferation and growth of follicles (Shah et al., 2018). In conclusion, electroacupuncture may regulate the gut microbiota, activate the PI3K-AKT pathway, inhibit oxidative stress responses, and thus affect the proliferation and development of disordered cells.

## 3 Conclusion and outlook

Amount of researches suggest an intricate connection between the gut microbiota and the brain. The gut microbiota-brain axis has always been considered as a bridge and connection between the gut microbiota and the brain, which can be achieved through the VN, hypothalamic-pituitary-adrenal axis, cytokines, and some specific neurotransmitters’ direct or indirect chemical pathways (Forsythe et al., 2016; Morais et al., 2021). Currently, amount of researches indicate that the VN plays an important role in the gut microbiota-brain axis. The VN is composed of 80% afferent fibers and 20% efferent fibers, with afferent fibers sensing various gut information, then entering the nucleus tractus solitarius together with the VN, and projecting to the central nervous system, producing different behaviors and effects (Bonaz et al., 2018). A study involving a strain of *Lactobacillus* suggests that they can affect how certain brain receptors work in areas like the prefrontal cortex, amygdala, and hippocampus. Interestingly, when these bacteria were studied in mice with a specific surgical procedure (SDV), the effect on brain receptors was not seen (Bravo et al., 2011). Another study showed that SDV could stop behaviors that resemble depression and restore a healthy balance of gut bacteria in mice treated with LPS, supporting the idea that VN procedure can disrupt the connection between gut bacteria and the brain (Zhang et al., 2020). Furthermore, the incision of the subphrenic artery in the rats vaccinated with *Salmonella typhimurium* weakened c-fos expression in paraventricular nucleus (PVN) (Wang et al., 2002). Despite the fact that *Salmonella typhimurium* infection was associated with intestinal inflammation, subsequent studies have shown that even in the absence of a defined immune response, gut microbiota can directly activate neural pathways (Goehler et al., 2005). Researchers showed that the specific type of bacteria called *L. johnsonii* La1, when administered directly into the duodenum, can lower the activity of the sympathetic nervous system in the kidneys and decrease blood pressure, while stimulating the activity of the VN in the stomach (Tanida et al., 2005). These findings highlight the important function of VN fibers in facilitating communication between the gut microbiota and the brain.

Researchers have found that acupuncture at different acupoints, rather than invasive direct stimulation of the VN, can also produce strong anti-inflammatory effects. Researches over the past five years has shown that acupuncture completely or partially inhibits systemic inflammation through activating the VN. Acupuncture signals are mainly transmitted to the dorsal vagal complex, including the NTS and PVN (Lim et al., 2016). After integration of information in the brain, the acetylcholine (Ach) system is activated, including ACh and its receptors, acetyltransferase (ChAT) and AChE, and the cell JAK2/signal transducer and activator of transcription 3 (STAT3), NF-κB and MAPK pathways are modulated to achieve anti-inflammatory effects (Jie et al., 2018; Wang et al., 2018a; Zhang et al., 2018; Cao et al., 2021). At the same time, acupuncture signals from ST36 also activate the vagus-adrenal medullary-dopamine pathway, stimulating the release of dopamine from the adrenal glands, acting on DA type 1 receptors, and inhibiting cytokine production (Torres-Rosas et al., 2014; Li et al., 2021b). Acupuncture stimulates the body’s sensory signals at the activated acupoints and transmits these sensation signals to the spinal cord, brainstem, and hypothalamus neurons. After the brain integrates the information, it further stimulates various neuro-immune pathways, involving the VN-adrenal medulla-dopamine, cholinergic anti-inflammatory, sympathetic nerve pathways, as well as the hypothalamic-pituitary-adrenal axis, fully releasing key neurotransmitters and hormones to act on various cells and microenvironments of the body, thus participating in the occurrence and development of various diseases (Zhao, 2008; Browning et al., 2017; Ulloa et al., 2017; Park and Namgung, 2018; Dou et al., 2021).

This review describes the effect of acupuncture on various diseases through the regulation of gut microbiota. Gut microbiota is regarded as the “brain-gut organ” in modern medicine, and as research progresses, several diseases have been found to be closely related to gut microbiota. Acupuncture is a pivotal therapy used in traditional Chinese medicine that medical professionals and patients widely accepted because of its significant therapeutic effects, safety, and environmental friendliness. However, current research on the effects of acupuncture on gut microbiota has mainly focused on a limited number of diseases. The effects of acupuncture on gut microbiota are relatively singular, and research methods are mostly focused on direct microbial cultivation, with a narrow range of applicability and limited information acquisition. Therefore, future research should include more clinical studies and exploration of acupuncture’s effects on the VN mechanism, to investigate potential mechanisms through which acupuncture-induced modulation of gut microbiota influences different medical conditions, and to explore the role of the VN in the acupuncture influence on gut microbiota, and gather more evidence to support its early intervention as a standard treatment.

## Author contributions

HZ: Supervision, Writing – review & editing. HX: Writing – original draft. YL: Writing – original draft. QL: Writing – review & editing.
